# Impact of whole-herd vaccination on a caprine coxiellosis outbreak: a longitudinal study of *Coxiella burnetii* shedding, serology, and host microbiota

**DOI:** 10.3389/fmicb.2026.1824800

**Published:** 2026-06-03

**Authors:** Raquel Toledo-Perona, Jesús Gomis, Nerea Bailon-Larrañaga, Antonio Contreras, Juan José Quereda, Pedro González-Torres, Nerea Carrón, Ángel Gómez-Martín

**Affiliations:** 1 Research Group Microbiological Agents Associated with Animal Reproduction (ProVaginBIO), Departamento Producción y Sanidad Animal, Salud Pública Veterinaria y Ciencia y Tecnología de los Alimentos, Facultad de Veterinaria, Universidad Cardenal Herrera-CEU, CEU Universities, Alfara del Patriarca, Valencia, Spain; 2 Department of Animal Health, Faculty of Veterinary Medicine, Universidad de Murcia, Murcia, Spain; 3 Research Group Listeria: Biology and Infection (LisBio), Departamento Producción y Sanidad Animal, Salud Pública Veterinaria y Ciencia y Tecnología de los Alimentos, Facultad de Veterinaria, Universidad Cardenal Herrera-CEU, CEU Universities, Alfara del Patriarca, Valencia, Spain; 4 Microomics Systems S.L., Barcelona, Spain

**Keywords:** Q fever, abortion, dysbiosis, metabarcoding, ruminants

## Abstract

This longitudinal study investigated *C. burnetii* dynamics and the associated microbiota after vaccination in aborted and normal-delivery goats and bucks following a Q fever outbreak. Fecal, milk, vaginal, preputial, nasopharyngeal, environmental (qPCR and *16S rRNA*), and blood samples (*16S rRNA* and serology) were analyzed. Before vaccination, over 70% of animals were seropositive, with qPCR positivity in all females and half of the males. Seroconversion remained complete until the second kidding season and reached approximately 90% by the third kidding season. After primary vaccination, a significant 60% reduction in shedders was observed, with shedding remaining absent during the two consecutive kidding seasons. Nasopharyngeal samples showed the highest and most persistent positivity, highlighting their diagnostic and epidemiological relevance. The first detection of *C. burnetii* in the buck prepuce underscores the potential role of males in disease maintenance. Persistent environmental contamination further supports early, whole-herd vaccination as a long-term strategy. Metabarcoding revealed significant shifts in microbial diversity across all anatomical sites over time, including vaginal community structure, suggesting local dysbiosis after abortion. The DNA detection of *C. burnetii* in blood and its significantly higher vaginal abundance in aborted females may contribute to abortion. A reduction in vaginal pathogen load (*p* < 0.05) may suggest a reproductive microbiota modulation associated with increased Firmicutes, considering it as a potentially health biomarker.

## 1 Introduction

*Coxiella burnetii* (*Cb*) is the causative agent of the zoonosis Q fever. Small ruminants are considered the main reservoir of Cb and the primary source of human infection (Schneeberger et al., 2014; van den Brom et al., 2015a; Pexara et al., 2018), being responsible for the majority of human Q fever outbreaks occurred (Roest et al., 2011; Boarbi et al., 2014; Anderson et al., 2015; Avberšek et al., 2019; Pouquet et al., 2020). Transmission between animals and humans primarily occurs via airborne exposure (Maurin and Raoult, 1999) through inhalation of the bacteria (Angelakis and Raoult, 2010). Although *Cb* can be asymptomatic (Arricau-Bouvery and Rodolakis, 2005), late-term abortion is the most common sign in sheep and goats, often accompanied by weak premature neonates, or less frequently metritis in caprine (van den Brom et al., 2015a). Respiratory disorders in sheep (Martinov et al., 1989) or the negative impact on milk quality and production, especially in dogs (Canevari et al., 2018), have also been described. Goats are generally more susceptible to coxiellosis than sheep, frequently experiencing more severe abortion storms affecting up to 90% of pregnant females, and exhibiting higher seroprevalence (van den Brom et al., 2015a; Álvarez-Alonso et al., 2018; Bauer et al., 2020). Moreover, some goats develop chronic disease, possibly associated with bacterial colonization of tissues such as the mammary gland (Bauer et al., 2024) or the genital tract (Alsaleh et al., 2011), thereby enabling the herd to remain persistently infected (Berri et al., 2007).

Milk, vagina secretions, and feces are described as the main bacterial shedding routes in goats (Rodolakis et al., 2007, 2009). Recent evidence of high *Cb*-positivity and diagnostic sensitivity in nasal samples suggests an underestimated respiratory tropism in goats (Toledo-Perona et al., 2025). Massive bacterial shedding into the environment primarily occurs after delivery or abortion (Todkill et al., 2018; Álvarez-Alonso et al., 2020; Zendoia et al., 2024) and can persist for several months in symptomatic or asymptomatic females (Roest et al., 2013; Joulié et al., 2015; van den Brom et al., 2015a) in different body tissues and fluids (Rodolakis, 2009). Despite bacterial shedding naturally decreasing over time (Berri et al., 2002; Miller et al., 2024), without control measures, environmental contamination and shedding from goats may persist for up to 4 years (Miller et al., 2024). Bacterial shedding dynamics after Q fever outbreaks in goats have demonstrated a vaginal *Cb* elimination for up to 3 months, 5 months in feces, and between 1 and 4 months in milk (Roest et al., 2012; Álvarez-Alonso et al., 2018; Miller et al., 2024). Further to the aforementioned, abortion episodes may recur over several consecutive kidding seasons (Berri et al., 2005; EFSA, 2010; Miller et al., 2024; Zendoia et al., 2024). Moreover, recent findings of *Cb* detection in rams and bucks (Ruiz-Fons et al., 2014; Wolf et al., 2020; Sánchez-Rodríguez et al., 2024; Toledo-Perona et al., 2025) support a potential Q fever epidemiological contribution of males and the presence of *Cb* in the preputial microbiota of bucks from infected herds.

Due to the bacteria’s high resistance to adverse environmental conditions (Arricau-Bouvery and Rodolakis, 2005), they can remain viable in dust samples for over four kidding seasons (Álvarez-Alonso et al., 2020). Previously unrecognized environmental sources of *Cb* contamination have recently been described, such as feeding troughs (Toledo-Perona et al., 2025), emphasizing the need to identify additional contamination points in livestock herds. The most common measures against coxiellosis in ruminant herds are vaccination together with hygienic and biosecurity measures (EFSA, 2010; Mori and Roest, 2018; Gunther et al., 2019; Toledo-Perona et al., 2024). With respect to vaccines, the inactivated phase I vaccine is the only authorized against *Cb* for domestic ruminants (Gisbert et al., 2024). Related to vaccine control efficiency, some authors described a reduction in bacterial shedding (Rousset et al., 2009a; Sting et al., 2013; Boarbi et al., 2014; Bauer et al., 2022a; Zendoia et al., 2024) and clinical signs after vaccination in goats (Hogerwerf et al., 2011), along with the reduction of the coxiellosis prevalence infection in caprine flocks (van den Brom et al., 2015b). Longitudinal studies have evaluated the impact of the vaccine in dairy goats (Álvarez-Alonso et al., 2018; Bauer et al., 2022b; Zendoia et al., 2024) and estimated that the control of Cb-infection may be accomplished in 2–7 years (van Asseldonk et al., 2015; Bontje et al., 2016; Zendoia et al., 2024). Vaccinating only yearlings is considered an ineffective control strategy against coxiellosis, as shedding may persist for 3–9 years post-vaccination, suggesting an early and whole-herd vaccination in small ruminant herds (Böttcher et al., 2022; Jansen et al., 2022; Zendoia et al., 2024). Consequently, long-term studies are needed to assess excretion and seroconversion dynamics in fully vaccinated goat herds after a Q fever outbreak.

In addition to serology and PCR (Rousset et al., 2009b; de Cremoux et al., 2012a; Toledo-Perona et al., 2024), *16S rRNA* sequencing datasets enable multi-population exploration of Q fever-associated microbiota. In this context, the impact of *Cb* on the bacterial communities of aborted and normal-delivery sheep and goats (Toledo-Perona et al., 2025) was highlighted. Metabarcoding allows us to describe bacterial patterns in different anatomic locations, identifying novel populations in not commonly found sites, taxa linked to health and fertility status, and even female–male microbiota modulation (Serrano et al., 2020; Reinoso-Peláez et al., 2023; Barba et al., 2024; Toledo-Perona et al., 2025). To date, a single metabarcoding study in Cb-infected small ruminants has revealed microbiota alterations, including a vaginal taxonomic profile linked to infertility in aborted females (Toledo-Perona et al., 2025).

To the best of the author’s knowledge, shedding patterns, seroconversion, and their associated host microbiota changes in naturally *Cb*-infected goats and bucks have not been individually evaluated over time after an early full-herd vaccination following a Q fever outbreak. Finally, the evolution of the whole caprine microbiota after a Q fever outbreak remains unknown. Accordingly, the main hypothesis of the present study is that fluctuations in the diversity and composition of the caprine microbiota could occur after an outbreak of Q fever. In addition, early entire-herd vaccination could reduce the time to detect *Cb* in animals and environmental samples. Therefore, the objective of the observational study was to monitor *Cb* presence and associated microbiota over three kidding seasons using qPCR and metabarcoding of environmental and animal samples, alongside individual serological monitoring of a goat herd following a Q fever outbreak and full-herd vaccination.

## 2 Materials and methods

### 2.1 Study population description

The selected herd, located in southern Spain, was composed of 250 animals in a semi-intensive dairy system. This flock used to purchase replacement animals from external farms. At the study onset, high abortion rates (> 60%) and an elevated percentage of vaginal samples only positive to *Cb* by PCR were observed during the kidding season. In many goats, other clinical signs were noted irrespective of abortion, such as nasal discharges, apathy, anorexia, and fever. A Q fever outbreak with high abortion rates occurred 5 years after the introduction of new animals. Finally, although a *Chlamydia abortus* vaccination program had been implemented, a coxiellosis vaccination strategy had not yet been introduced. Based on the available epidemiological data, the herd was consequently included in the present longitudinal Q fever vaccination study.

Regarding control, hygienic and biosecurity measures implemented after the initial outbreak, the farmer introduced manure removal, improved the management of potentially infectious material, such as placentas, and avoided the introduction of new replacement animals into the herd.

### 2.2 Sampling approach

The study protocol was reviewed and approved by the Animal Welfare & Ethics Committee of CEU Cardenal Herrera University (Alfara del Patriarca, Spain) and the Spanish Regional Government Generalitat Valenciana (Alfara del Patriarca, Spain; 2024-VSC-PEA-0120).

**Figure 1:**
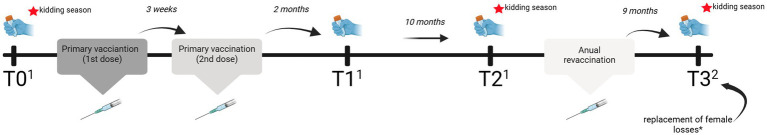
Sampling time points and vaccination schedule performed for serological and molecular analysis (qPCR and metabarcoding). T0 = during the first week after abortion or delivery, before the primary vaccination; T1 = 2 months after the second dose of primary vaccination; T2 = within 1 week postpartum of the next kidding (10 months after the primary vaccination and before revaccination); T3 = within 1 week postpartum of the next kidding (9 months after revaccination). ^1^Serology, qPCR and metabarcoding analysis; ^2^Serology and qPCR analysis. *Due to mortality or culling (*n* = 10), an equal number of females from the same herd were introduced as replacements at T3. Created in BioRender.com.

The sampling schedule was divided in different time points: T0, during the first week after abortion or delivery, before the primary vaccination; T1, 2 months after the second dose of primary vaccination; T2, within 1 week postpartum of the next kidding (10 months after the primary vaccination and before revaccination); T3, within 1 week postpartum of the next kidding (9 months after revaccination; Figure 1). At T0, after the alarming abortion rates, no antibiotic treatment was applied to symptomatic animals included in the study. An individual monitoring of 18 goats (16 females and two males) was performed at T0 and T1. At T2, due to animal death or replacement, only six monitored females remained in the herd. At the first three time sampling points, were established by randomly selecting goats that had either delivered or aborted within the same kidding season: G1, group 1 (aborted females); G2, group 2 (normal-delivery females that underwent a successful delivery). Males were included in the G3 group, which was used for natural mating. Finally, in order to evaluate the seropositivity and bacterial shedding during the second vaccination year (T3), the original goats were replaced with new females of similar age and vaccination status, using the same males. Newly introduced goats were part of the flock at the time of the abortion outbreak and were housed together with the other study animals. At all time points, all females were kept in the same pen over the study. The total herd vaccination using a phase I inactivated vaccine was carried out after the outbreak, following the manufacturer’s instructions (Coxevac®, Ceva Santé Animale, Libourne, France). Sampling was performed during the spring season. No dietary modifications were implemented during the course of the study.

Environmental samples from the milking parlor, feeding trough swabs, and milking machine swabs from the inner surface of rubber hoses that transport milk from the collector to the stainless-steel collection tube, which showed signs of wear and biofilm presence, were taken, along with a sample from a fly trap covered by dust. In addition, bedding samples from the kidding area and from domestic animals (chicken and dog feces, recent soil-derived, this last introduced to the farm at T1) were sampled due to their proximity and direct contact with goats. The statistical differences between groups and times regarding positive qPCR samples and seropositivity were studied with the EpiInfo software (Dean et al., 2011) using chi-square correction (Yates) with a 95% confidence level.

For sample collection, at least two researchers assisted in restraining the animals gently for the sample collection and were equipped with appropriate personal protective equipment (Toledo-Perona et al., 2025). Environmental samples and blood, feces, individual raw milk, along with nasopharyngeal and vaginal/preputial swabs were obtained for metabarcoding analysis. All these same samples, except blood, were also collected to detect *Cb* DNA by qPCR. A serological study was performed using blood samples (Vacutainer® SST, 5 mL serum separation tube (Bauer et al., 2020; Toledo-Perona et al., 2025). Before blood collection, the skin was shaved and disinfected with 2% chlorhexidine solution. For the molecular study of genital and respiratory samples, swabs were obtained with a sterile DNA-free cotton swab (Deltalab®-ref. 300,263) for metagenomic analysis and an AMIES PS + VISCOSA swab (Deltalab®-ref. 300,287) for qPCR diagnosis. After cleaning and disinfection with 2% chlorhexidine solution, swab samples were obtained by gently swabbing the internal mucosa of the deep vagina, preputial sac, and nasopharynx (Nugeyre et al., 2019; Wickware et al., 2020; Barba et al., 2024). Environment samples, including surfaces from feeding troughs, the milking machine components mentioned above, and chicken feces, were sampled using swabs (Toledo-Perona et al., 2025). Following the methodology of Toledo-Perona et al. (2025) for dairy and fecal samples, milk sampling was preceded by teat cleaning and disinfected with 70% alcohol, dried with sterile gauze, and the first milk fraction was removed. Fecal samples were collected directly from the rectum using sterile gloves. Feces from dogs and the fly trap dust were collected directly using sterile gloves. Moreover, bedding samples from the delivery zone location were also taken (Avberšek et al., 2019; Toledo-Perona et al., 2025). Sterile cryovials (Deltalab®-ref. 409106.1) were used for feces, milk, and environmental samples as described in previous studies (Mamun et al., 2020; Biçer et al., 2021). Samples were stored at −20 °C for qPCR and serologic analysis, and at −80 °C for metabarcoding analysis. A total of 62 blood samples were used for serologic analysis. For qPCR, 258 samples were obtained (62 nasopharyngeal swabs, 62 feces, 54 vaginal swabs, 54 individual milk, eight preputial swabs, four trough swabs, four bedding samples, four domestic chicken fecal swabs, three surfaces swabs from the rubber hoses of the milking machine, a fly trap sample, and one fecal dog’s sample). For metabarcoding analysis, 219 samples were analyzed, including the same type of animal samples and environmental samples corresponding to T0, T1, and T2 in order to performed an individual microbiota evolution over time (feeding trough swabs, milking machine swabs, bedding samples, domestic chicken fecal swabs, and two fecal samples from dog) used for qPCR diagnosis, as well as 36 blood samples for serological analyses.

### 2.3 Serological (enzyme-linked immunosorbent assay) and molecular (qPCR) analyses

To evaluate seropositive animals against *Cb*, an ELISA TEST (enzyme-linked immunosorbent assay) was performed in serum samples (*Coxiella burnetii* Monoscreen Ab-ELISA. BIO-X DIAGNOSTICS® K 298/2) following the procedure previously described (Toledo-Perona et al., 2025). Following the manufacturer’s instructions, for the results interpretation, the coefficient (S/P %) was calculated for each sample using the formula:

$$\begin{array}{l} (\%)\;S/P=\mathrm{OD}\;\text{sample}-\mathrm{OD}\;\text{negative serum}\\ /\mathrm{OD}\;\text{positive serum}-\mathrm{OD}\;\text{negative serum}*100. \end{array}$$

The ratio ELISA-Ac IgG was interpreted: Negative samples = (%) S/*p* < 40%; doubtful samples = 40% ≤ (%) S/*p* ≤ 60%; positive samples = (%) S/*p* > 60%.

DNA was extracted using a commercial kit (MagMAX CORE Nucleic Acid Purification kit, Applied Biosystems, Thermo Fisher Scientific®, Ref. A32702) from swabs, fecal, milk, and organic environmental material following the manufacturer’s instructions for a low-input workflow previously described (Toledo-Perona et al., 2025). After sample preparation, using the Automated Nucleic Acid Purification System Zixpress 32 (Zinexts Life Science Corporation), they were processed according to the manufacturer’s instructions for the extraction kit. The presence of *Cb* DNA was investigated by a real-time PCR procedure targeting the transposon-like repetitive region *IS1111* of the *Cb* genome. qPCR was performed by a commercial kit (*Coxiella burnetii* monodose DTEC-qPCR with internal control, GPS Genetic Analysis Strategies®). Samples with a Ct < 37 were considered positive, while those with a Ct ≥ 37 were considered negative.

### 2.4 Metabarcoding analyses

#### 2.4.1 Library preparation and sequencing

The composition and structure of microbial communities were assessed using a marker-based approach, the 16S ribosomal RNA subunit gene (*16S rRNA*). An amplification and sequencing of the V3–V4 regions of the *16S rRNA* gene, following previously published studies (Barba et al., 2024). DNA extraction of swab, blood, and milk samples was performed following a methodology previously described (Barba et al., 2024) using a commercial kit DNeasy PowerLyzer PowerSoil (Qiagen, Hilden, Germany). DNA extraction of fecal and environmental samples was performed using a commercial kit (MagMAX CORE Nucleic Acid Purification Kit, Applied Biosystems, Thermo Fisher Scientific®, Ref. A32702) with the extraction equipment (ZIXpress 32, Zinexts Life Science Corporation). Sequencing was carried out using the Illumina MiSeq platform with 2 × 300 bp paired-end reads and v3 chemistry, applying a loading concentration of 10 pM (Barba et al., 2024; Toledo-Perona et al., 2025). As part of the library preparation and quality control procedures, a defined mock community DNA was incorporated as a positive control (Zymobiomics Microbial Community DNA, Catalog Nos. D6305, Zymo Research, Irvine, CA, United States), together with two negative controls comprising a DNA extraction blank and an amplification blank. The quality ratios obtained (260/230 and 260/280) and DNA concentration levels after the extraction were optimal for this type of sample. Total DNA concentrations exceeded 10 ng/μL in a substantial proportion of samples, with all samples exhibiting concentrations within or above the 5–20 ng/μL range. The use of negative controls allowed for monitoring the library preparation and extraction process in low-concentration samples. PCR products were visualized by electrophoresis on a 1.5% agarose gel stained with SYBR Safe (Applied Biosystems, Thermo Fisher Scientific, Waltham, MA, USA), showing no visible bands in the negative controls. Amplification was performed over 25 PCR cycles for all sample types, including fecal, swab, milk, and blood samples (Barba et al., 2024; Toledo-Perona et al., 2025). The PCR included: 3 min at 95 °C (initial denaturation) followed by 25 cycles: 30 s at 95 °C, 30 s at 55 °C, and 30 s at 72 °C, and a final elongation step of 5 min at 72 °C. PCR products were purified using AMPure XP beads (Beckman Coulter, Nyon, Switzerland) with a 0.9 × ratio according to the manufacturer’s instructions.

#### 2.4.2 Bioinformatics processing and analysis

Two metabarcoding comparisons were performed. The first objective was to describe the microbiota and assess differences between female groups (G1 *vs.* G2) over time (T0 *vs.* T1). Moreover, a microbiota description and comparison between T0 and T1 were performed for male and environmental samples. Given the heterogeneous origin of environmental samples, the T0-T1 comparison was conducted irrespective of matrix. The second metabarcoding comparison evaluated the differences and described the microbiota between females 10 months after primary vaccination (T2), as well as the metabarcoding results from males and environmental samples.

Raw demultiplexed forward and reverse reads were processed using QIIME2 version 2020.11 with default parameters unless stated (Bolyen et al., 2020). For the quality filtering, denoising, pair-end merging, and Amplicon Sequence Variants (ASVs) assignation, DADA2 (Callahan et al., 2016) was used. Phylotype data were used to calculate the following alpha diversity metrics: community richness (observed ASVs) and evenness (Pielou’s evenness index). Alpha diversity comparisons were performed using a Generalized Linear Mixed Model; the R package NBZIMM v.1.0 (Zhang and Yi, 2020) was used for richness, and the R package betareg v.3.1–4 (Cribari-Neto and Zeileis, 2010) for evenness were aligned using the qiime alignment mafft method (Katoh and Standley, 2013) in order to create a phylogeny fasttree to calculate phylogenetic relations between ASVs using qiime2 (Price et al., 2009). ASV tables were subsampled without replacement in order to have even sample sizes for diversity analysis using qiime diversity core-metrics-phylogenetic pipeline. The smallest sample size was chosen for subsampling (Lozupone and Knight, 2005). ASVs and phylogenetic data were used to calculate the following beta diversity metrics: Unweighted UniFrac, Weighted UniFrac, Jaccard, and Bray-Curtis. Beta diversity distance matrices were used to calculate principal coordinates analysis (PCoA) and to make ordination plots using the R software package version 4.2.0. The significance of groups was tested using Permanova and ANOSIM tests. Permdisp test was used to identify location vs. dispersion effects (Anderson and Walsh, 2013).

Taxonomic assignment of ASVs was performed using a Bayesian Classifier (Wang et al., 2007) trained with the Silva database version 138 (99% ASVs full-length sequences) using the qiime feature-classifier classify-sklearn method (Abraham et al., 2014). Differential abundance of taxa was tested using a Negative Binomial Generalized Linear Mixed Model. A significant threshold for all statistical analyses was set at 0.05. BiodiversityR version 2.14–1, PMCMRplus version 1.9.4, RVAideMemoire version 0.9–8, and vegan version 2.5–6 packages were used for the different statistical analyses carried out. The taxonomic profile of the mock community control matched the expected bacterial profile.

## 3 Results

### 3.1 Serological and qPCR results

**Figure 2:**
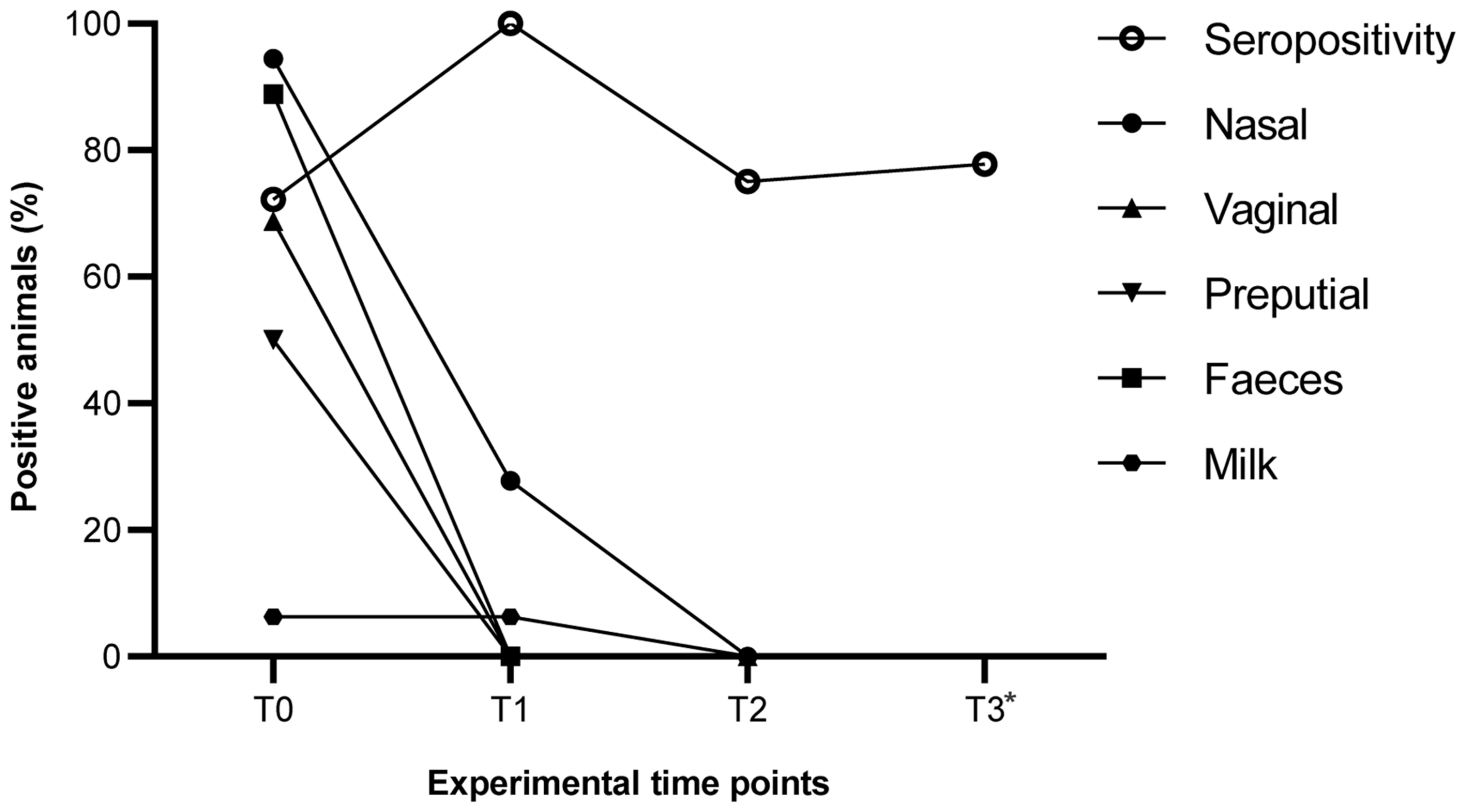
Seropositivity and positive qPCR animals included in the study over time. T0 = during the first week after abortion or delivery, before the primary vaccination; T1 = 2 months after the second dose of primary vaccination; T2 = within 1 week postpartum of the next kidding (10 months after the primary vaccination and before revaccination); T3 = within 1 week postpartum of the next kidding (9 months after revaccination). *Due to mortality or culling (*n* = 10), an equal number of females from the same herd were introduced as replacements at T3.

After T0, the farmer reported no abnormalities in abortion rates or other clinical signs during the next kidding seasons. Due to mortality and culling of females at T2, 10 goats were incorporated into the study population at T3 as replacements to assess seropositivity and *Cb* detection by qPCR. Individual qPCR results and seropositivity results of the monitored goats up to T2 are shown in Table 1. Results from T3 were excluded from Table 1 because newly enrolled goats were introduced at this time point. Figure 2 shows seropositive and qPCR-positive animals over time, including newly introduced females to the study population. Differences in serological results could not be analyzed due to the insufficient number of observations (< 5) for a valid Chi-square test. Previously to vaccination (T0), the seropositivity observed was 72% (13/18). Particularly, G1 females showed higher seropositivity (88%) than G2 (75%). At T1, all females and males were seropositive, and seropositivity was sustained in females until T2. Finally, after the introduction of new females (T3), 88% of females were seropositive. Males were seronegative at T0, T2, and T3. In the case of qPCR results, no significant differences between positive samples among female groups were observed. Nevertheless, qPCR positive samples from all animals decreased at T1 compared to T0 (*p* < 0.05). In that sense, all females and one buck presented *Cb*-positive samples (94%; 17/18) at T0. Nasopharyngeal swabs showed the highest detection rate of the pathogen in goats and bucks (94%; 17/18), followed by feces (89%; 16/18). Regarding genital samples, 100% of aborted and 38% of normal-delivery females presented positive vaginal samples (69%; 11/16), including one preputial sample. Finally, only one aborted female shed *Cb* by milk at T0 (6%; 1/16). After primary vaccination (T1), a total of 33% goats still tested positive in qPCR. Specifically, 38% of G1 (3/8) and 25% of G2 goats (2/8) had positive nasopharyngeal samples, including one positive milk sample (6%; Table 1). At T2 and T3, no positive animal samples by qPCR were detected.

**Table 1:** Individual serological and qPCR results for the original animals included in the study over time (T0, T1, and T2).

Animal group		G1 (aborted females)								G2 (normal-delivery females)								G3 (males)	
Animal		1	2	3	4	5	6	7	8	9	10	11	12	13	14	15	16	17	18
Serology	T0	+	+	+	+	+	+	+	−	+	+	+	−	+	−	+	+	−	−
	T1	+	+	+	+	+	+	+	+	+	+	+	+	+	+	+	+	+	+
	T2	ND	+	+	ND	ND	ND	+	ND	ND	+	ND	ND	+	ND	+	ND	−	−
Nasal	T0	+	+	+	+	+	+	+	+	+	+	+	+	+	+	+	+	+	−
	T1	+	−	−	−	+	−	+	−	−	+	−	−	−	+	−	−	−	−
	T2	ND	−	−	ND	ND	ND	−	ND	ND	−	ND	ND	−	ND	−	ND	−	−
Vaginal/Preputial	T0	+	+	+	+	+	+	+	+	+	−	−	−	−	−	+	+	+	−
	T1	−	−	−	−	−	−	−	−	−	−	−	−	−	−	−	−	−	−
	T2	ND	−	−	ND	ND	ND	−	ND	ND	−	ND	ND	−	ND	−	ND	−	−
Feces	T0	+	+	+	+	+	+	+	+	+	+	+	+	+	+	+	+	−	−
	T1	−	−	−	−	−	−	−	−	−	−	−	−	−	−	−	−	−	−
	T2	ND	−	−	ND	ND	ND	−	ND	ND	−	ND	ND	−	ND	−	ND	−	−
Milk	T0	−	−	−	+	−	−	−	−	−	−	−	−	−	−	−	−		
	T1	−	−	−	−	−	−	−	−	−	−	+	−	−	−	−	−		
	T2	ND	−	−	ND	ND	ND	−	ND	ND	−	ND	ND	−	ND	−	ND		

Only individuals tested from baseline were included (10 goats died or were culled from T2 onwards). T0 = during the first week after abortion or delivery, before the primary vaccination; T1 = 2 months after the second dose of primary vaccination; T2 = within 1 week postpartum of the next kidding (10 months after the primary vaccination and before revaccination); ND = no data.

In reference to environmental samples, qPCR results over time are shown in Table 2. At T0, domestic chicken’s feces, bedding from the kidding area, feeding troughs and fly trap samples from the milking parlor were positive. Due to the potential consideration of insect traps as sources of *Cb*-infection, the farmer decided to remove them from the milking parlor after T0. Bedding and feeding troughs remained positive at T1, along with swabs from the rubber hoses of the milking machine. Ultimately, all environmental samples at T2 and T3 were negative.

**Table 2:** Environmental samples result for qPCR analysis over time.

Time sampling points	T0	T1	T2	T3
Bedding sample	+	+	−	−
Feeding troughs^1^	+	+	−	−
Milking machine^1^^,^^2^	ND	+	−	−
Fly trap sample^1^	+	ND	ND	ND
Chicken feces swab	+	−	−	−
Dog’s feces sample^3^	ND	−	−	ND

T0 = during the first week after abortion or delivery, before the primary vaccination; T1 = 2 months after the second dose of primary vaccination; T2 = within 1 week postpartum of the next kidding (10 months after the primary vaccination and before revaccination); T3 = within 1 week postpartum of the next kidding (9 months after revaccination); ND = no data.

1 From milking parlor.

2 Rubber hose swabs.

3 New introduction at T1.

### 3.2 Metabarcoding results

The achieved sequencing depth and subsampling size were enough to observe the complete diversity present in the sample since a plateau was reached. Reads were truncated at the position when the 25th percentile Phred score fell below Q20: 293 bp for forward reads, and 228 bp for reverse reads. After quality filtering steps, merged and chimeral removal steps, the average sample size was 25,063 reads (min: 2,143 reads, max: 69,576 reads). Paired-end reads assigned to ‘Chloroplast’, ‘Mitochondria’, ‘d_Bacteria;’, ‘d_Eukaryota;’ and ‘Unassigned’ were removed. After quality filtering, trimming, and denoising steps, 2,300,913 filtered reads were used for phylotype calling DADA2 (Callahan et al., 2016). Singletons and doubletons were removed before diversity analysis. The total number of detected phylotypes in each sample, depending on sex and environmental samples, is shown in Supplementary Table S1. The results from the alpha and beta diversity analyses are described in Supplementary Tables S2, S3. With respect to the number of phylotypes in the comparison between T0 and T1, fecal samples in both sexes showed the highest number, followed by vaginal and preputial samples. In T2, fecal and milk samples presented the greatest number of phylotypes. Due to the insufficient number of reads in the case of male blood samples at T0-T1 comparison, bedding samples at T0, and vaginal samples at T2, as well as the reduced number of samples for all male samples at T2, it was not possible to perform statistical analyses (beta diversity) or to describe the bacterial taxonomy.

#### 3.2.1 Diversity analysis: alpha and beta diversity comparisons

**Figure 3:**
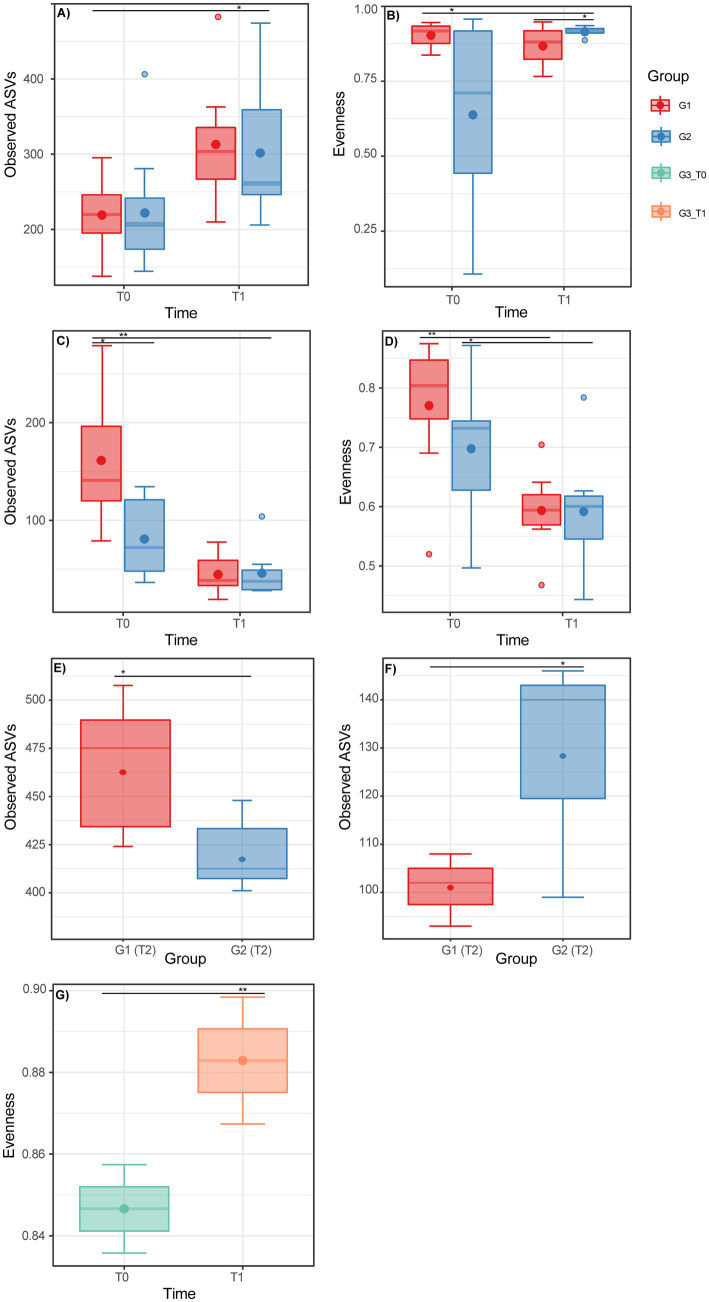
Significant differences in the alpha diversity (richness or evenness) for female (G1 = abortion females; G2 = normal-delivery females) and male (G3) samples. T0 = during the first week after abortion or delivery, before the primary vaccination; T1 = 2 months after the second dose of primary vaccination; T2 = within 1 week postpartum of the next kidding (10 months after the primary vaccination and before revaccination). *p-value < 0.05; **p-value < 0.01. **(A)** Fecal (T0-T1); **(B)** Milk (T0-T1); **(C)** Nasopharyngeal (T0-T1); **(D)** Nasopharyngeal (T0-T1); **(E)** Fecal (T2); **(F)** Nasopharyngeal (T2); **(G)** Preputial (T0-T1).

Richness and evenness indices were analyzed to assess differences across groups and times (Figure 3). Alpha diversity results showed significant differences between T0 and T1 in female fecal, milk, and nasopharyngeal samples, in addition to preputial samples. Regarding female samples, richness in nasopharynx and evenness index in milk and nasopharyngeal samples decreased at T1 compared to T0 (*p* < 0.05; Figures 3B–D), with significantly higher dairy microbiota uniformity in G2 than G1 at T1 (*p* < 0.05). On the contrary, female fecal richness (Figure 3A) and preputial microbial evenness (Figure 3G) increased after primary vaccination (T1; *p* < 0.05). At T2, fecal microbiota richness was higher in the G1 group (*p* < 0.05), whereas the nasopharyngeal microbiota was richer in G2 samples (*p* < 0.05; Figures 3E,F).

**Figure 4:**
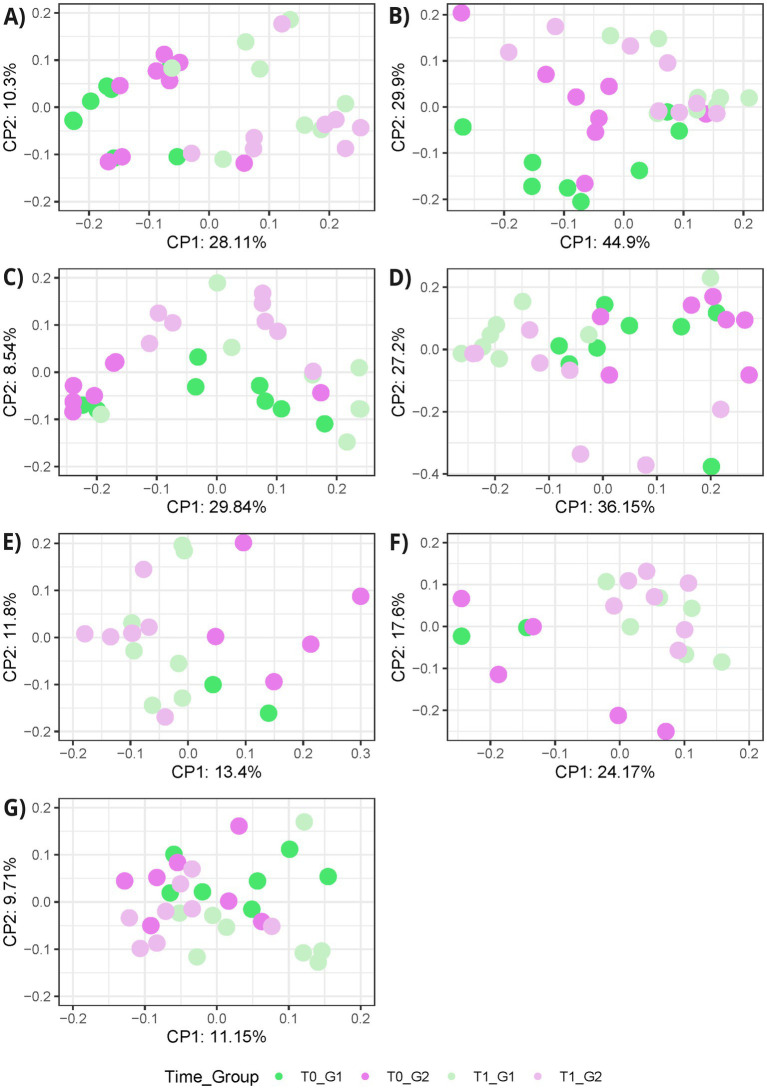
Significant differences in the beta diversity analysis in the comparison between female groups (G1 = abortion females; G2 = normal-delivery females) before and after primary vaccination. T0 = during the first week after abortion or delivery, before the primary vaccination; T1 = 2 months after the second dose of primary vaccination. **(A)** Nasopharyngeal samples (Unweighted Unifrac); **(B)** Nasopharyngeal (Weighted Unifrac); **(C)** Vaginal (Unweighted Unifrac); **(D)** Vaginal (Weighted Unifrac); **(E)** Blood (Unweighted Unifrac); **(F)** Blood (Weighted Unifrac); **(G)** Feces (Unweighted Unifrac).

PCoA based on Unweighted and Weighted Unifrac distances (PERMANOVA) revealed significant beta diversity differences in bacterial community structure across all anatomical locations, except milk, between G1 and G2 among T0 and T1 (Figure 4). In the case of nasopharyngeal (Unweighted and Weighted Unifrac Permanova, *p* < 0.01) and vaginal samples (Unweighted Unifrac Permanova, *p* < 0.05), both groups were segregated into different communities between T0 and T1 (Figures 4A–D). Moreover, vaginal microbiota showed significant differences between T0 and T1 for G1 (Unweighted and Weighted Unifrac Permanova, *p* < 0.05; Figures 4C,D). Finally, blood samples showed a lower dispersion result at T1 compared to T0 for G2 (Unweighted and Weighted Unifrac Permanova, *p* < 0.05; Figures 4E,F). Fecal, nasopharyngeal, vaginal, and blood samples were different between G1 at T0 and G2 at T1 samples, and the opposite (Permanova, *p* < 0.05). No structural differences were observed between male samples (T0-T1) or between female groups at T2. Similarly, environmental samples exhibited no significant differences in microbial diversity.

#### 3.2.2 General taxonomic composition: phylum description before and after primary vaccination (T0 and T1)

The V3–V4 region of the *16S rRNA* gene used in this study allowed the detection of both Bacterial and Archaeal communities. For this section, subsampling was not performed, and normalization of data was done either by calculating the relative abundance (RA) of reads or by using the offset term in the Generalized Linear Models (GLMs). Reads assigned to ‘Chloroplast’, ‘Mitochondria’, ‘d_Bacteria;’, ‘d_Eukaryota;’ and ‘Unassigned’ were removed from the analysis.

**Figure 5:**
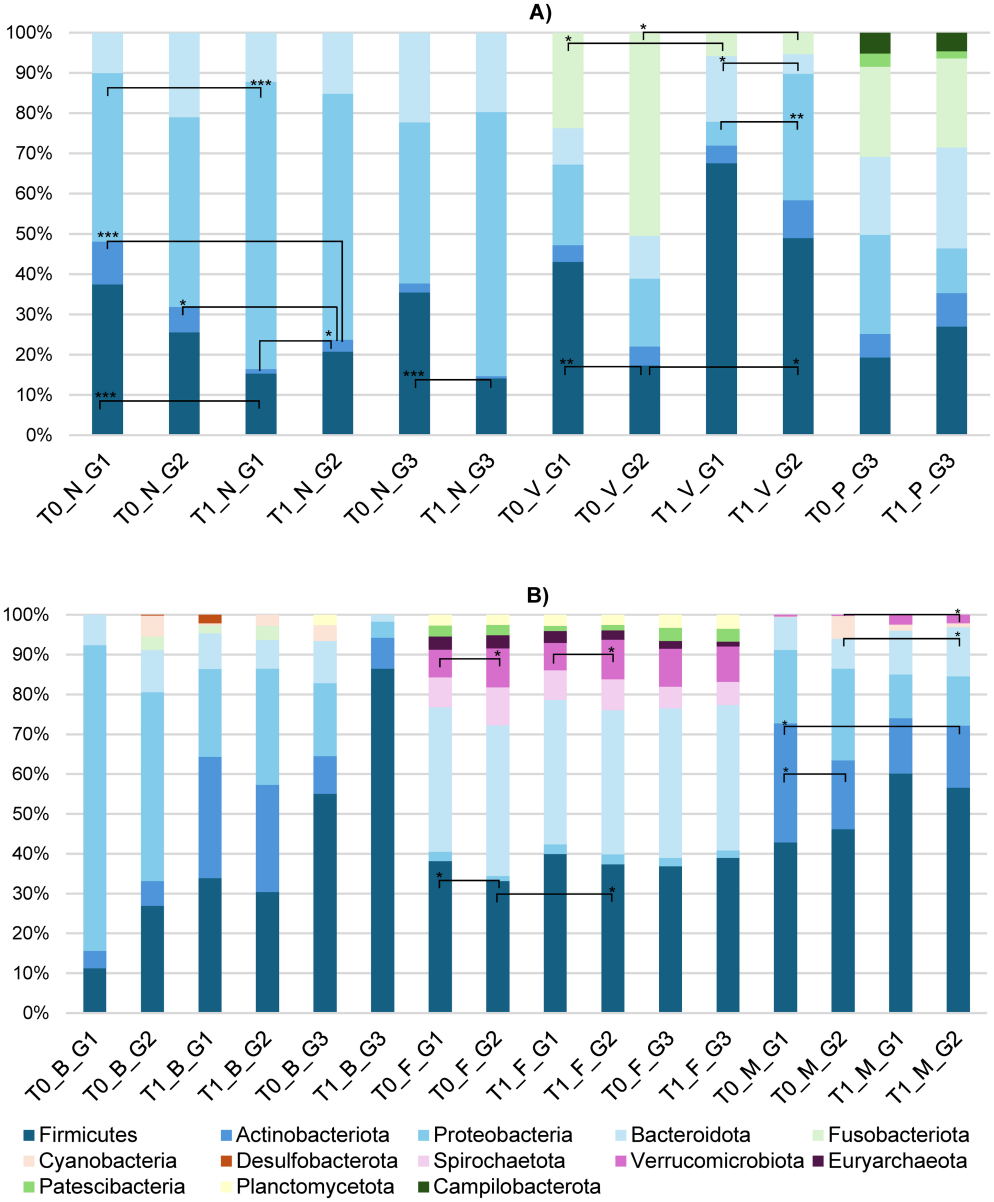
Relative abundance (RA) of taxa at the phylum level from female and male nasopharyngeal, vaginal/preputial **(A)**, blood, fecal and dairy samples **(B)** at T0 and T1. Only taxa with mean RA > 1% are shown. T0 = during the first week after abortion or delivery, before the primary vaccination; T1 = two months after the second dose of primary vaccination; G1 = group 1 (aborted females); G2 = group 2 (normal-delivery females); G3 = group 3 (males); B = blood; F = feces; M = milk; N = nasopharyngeal; V = vaginal; P = preputial. *p-value < 0.05; **p-value < 0.01; ***p-value < 0.001 (asterisk position indicates the most abundant phylum by group and time).

The phyla with the highest RA from each group and sample are described in Figure 5, including RA (%) significant differences (*p* < 0.05) between female groups in all anatomical locations except for blood microbiota, and for males in nasopharyngeal samples. Concerning the taxonomic composition of the blood microbiota, Proteobacteria was the most abundant phylum in male (T0 RA = 53%, T1 RA = 86%), along with female samples at T0 (RA = 54%). At T1, Firmicutes constitute the dominant phylum in the female blood microbiota (RA = 31%). In fecal samples, Bacteroidota presented the greatest RA at T0 for both sexes (RA = 36%). The phylum Firmicutes, which showed a significantly higher RA in G1 samples at T0 (*p* < 0.05), was the dominant taxa in the fecal microbiota of females (RA = 37%) and males (RA = 38%) at T1, with a significant increase observed in G2 samples (*p* < 0.05; Figure 5B). In addition, Verrucomicrobiota RA was statistically higher in G2 fecal samples at both time points (*p* < 0.05; Figure 5B). Regarding milk, Firmicutes was the dominant taxa over time (T0 RA = 43%, T1 RA = 56%). In the case of the nasopharyngeal microbiota, Proteobacteria exhibit the greatest RA in females (T0 RA = 49%; T1 RA = 40%) and males (T0 RA = 76%, T1 RA = 65%). In those samples, Firmicutes RA decreased significantly in G1 samples at T1 (*p* < 0.01), and Actinobacteria RA decreased over time for G2 samples, being higher than in G1 microbiota at T1 (*p* < 0.05; Figure 5A). In vaginal and preputial samples, Fusobacteria in the vagina (RA = 37%) and Proteobacteria in the prepuce (RA = 25%) showed the highest RA at T0, which reduced their RA in both female groups at T1 (*p* < 0.05; Figure 5A). At T1, Firmicutes dominated the microbiota in females (RA = 56%) and males (RA = 27%), with a significant increase in G2 samples (*p* < 0.05; Figure 5A).

#### 3.2.3 General taxonomic composition: genus and species description before and after primary vaccination (T0 and T1)

**Figure 6:**
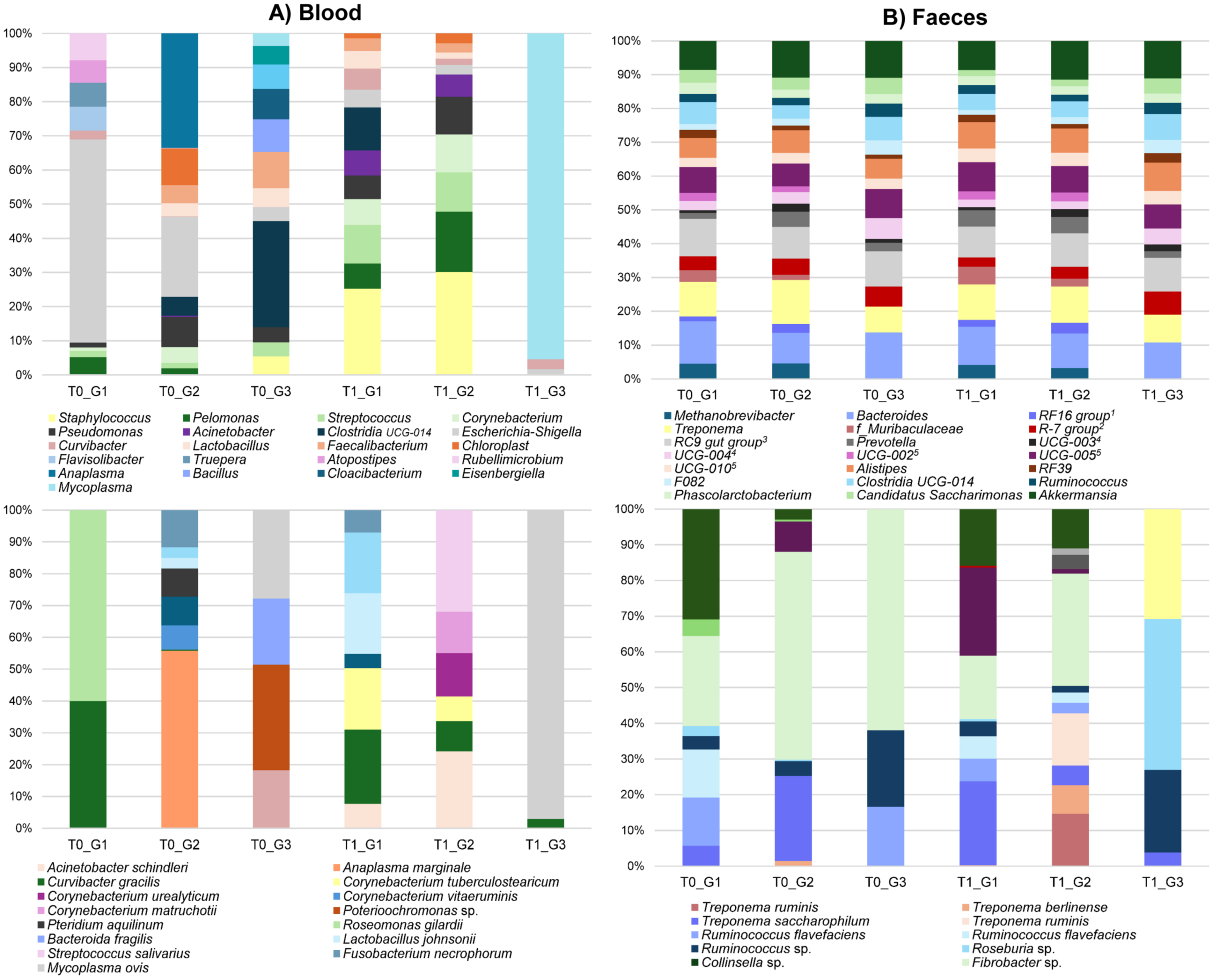
Relative abundance (RA) of taxa at the genus and species level of blood **(A)** and feces **(B)** samples from female and male samples at T0 and T1. Only taxa with mean RA > 1% are shown. T0 = during the first week after abortion or delivery, before the primary vaccination; T1 = 2 months after the second dose of primary vaccination; G1 = group 1 (aborted females); G2 = group 2 (normal-delivery females); G3 = group 3 (males). *f* = family. ^1^Bacteroidales family; ^2^Christensenellaceae family; ^3^Rikenellaceae family; ^4^Prevotellaceae family; ^5^Oscillospiraceae family.

**Figure 7:**
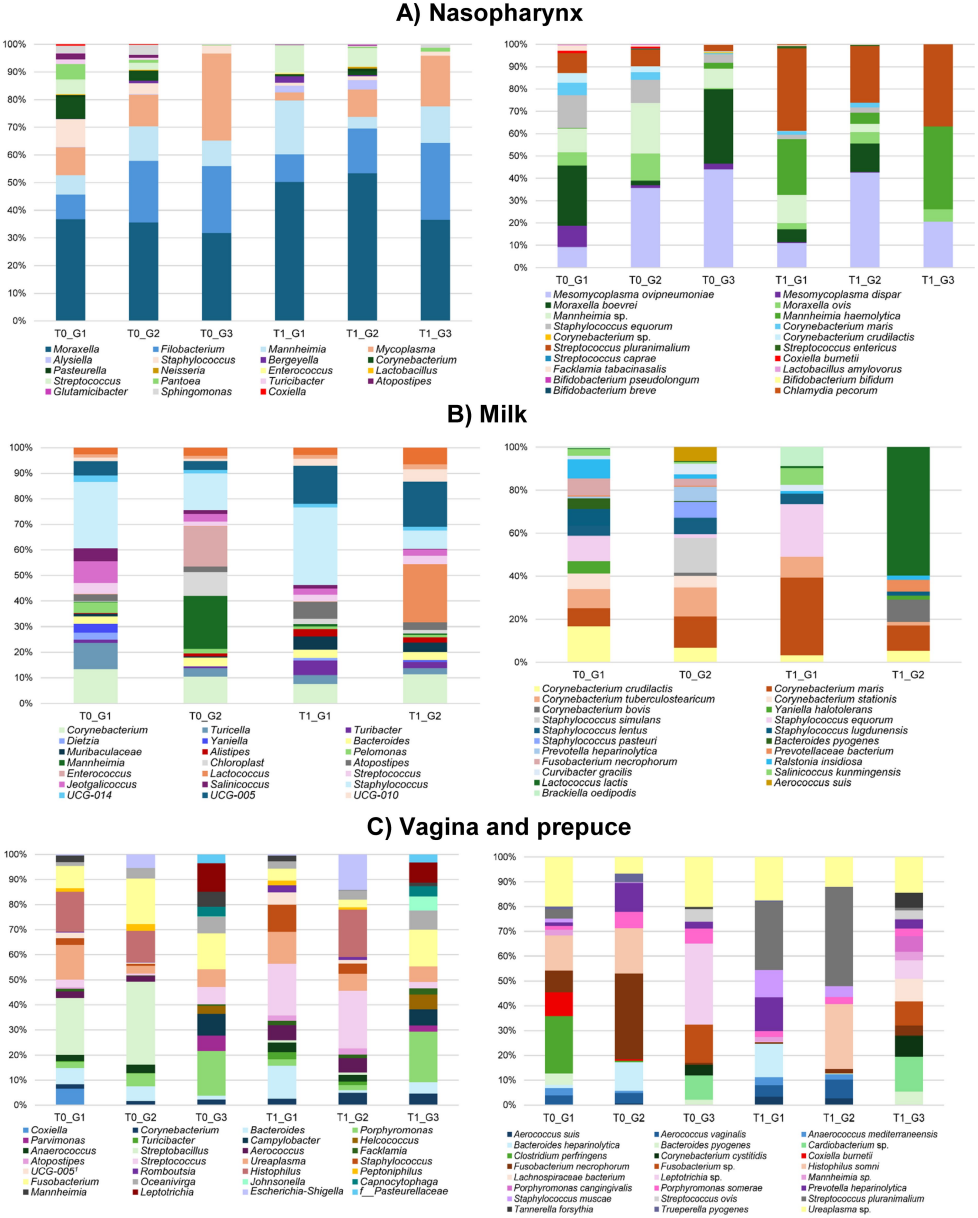
Relative abundance (RA) of taxa at the genus and species level of nasopharyngeal **(A)**, milk **(B)**, vaginal and preputial **(C)** samples at T0 and T1. Only taxa with mean RA > 0.50% (nasopharynx), 1% (milk), and 1.5% (vagina and prepuce) for the genera, and > 0.01% (nasopharynx), 0.20% (milk), and 1% (vagina and prepuce) for species are shown. T0 = during the first week after abortion or delivery, before the primary vaccination; T1 = 2 months after the second dose of primary vaccination; G1 = group 1 (aborted females); G2 = group 2 (normal-delivery females); G3 = group 3 (males). *f* = family. ^1^Oscillospiraceae family.

**Figure 8:**
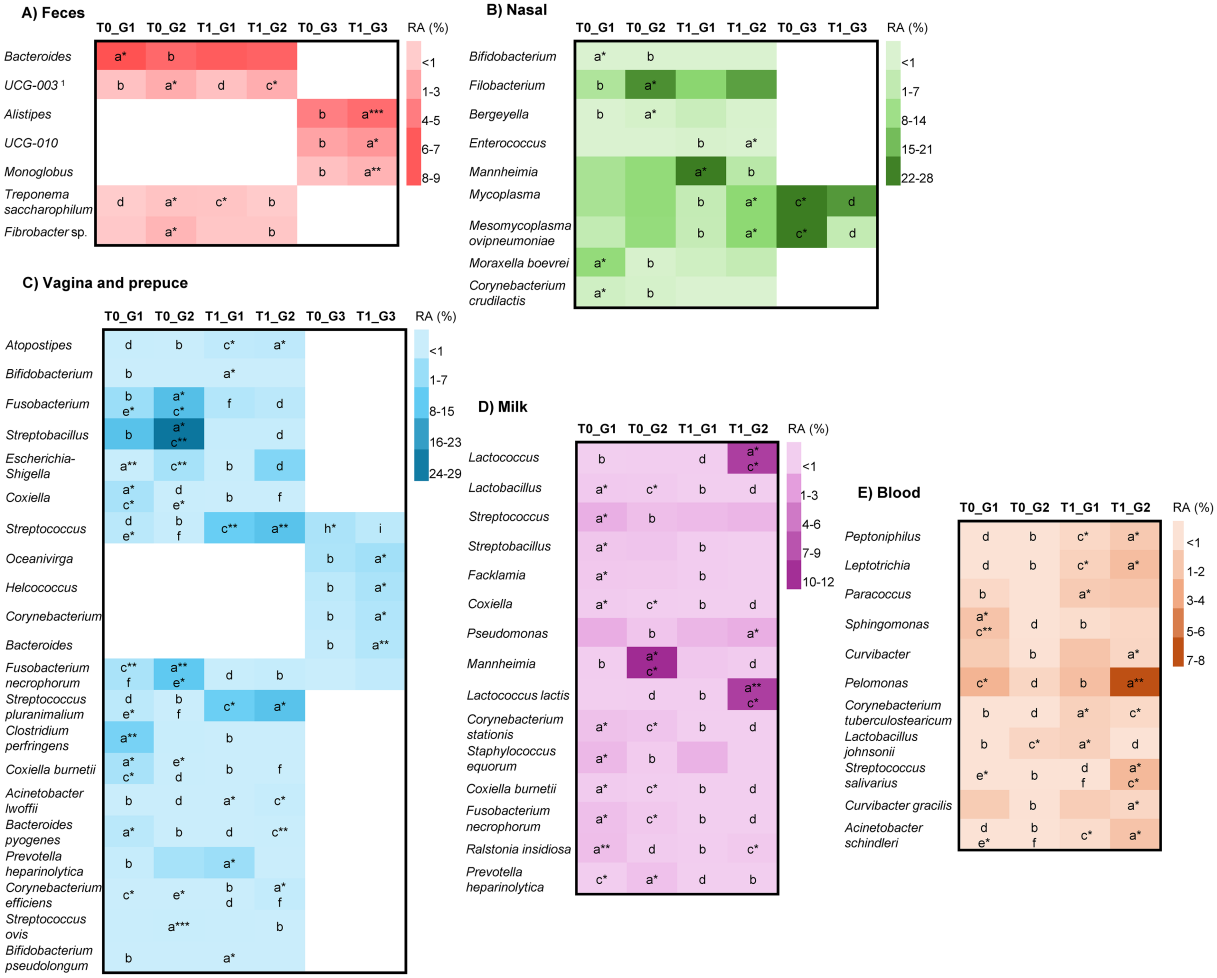
Significant differences in bacterial genera and species relative abundance (RA %) across female groups and males over time (T0-T1) according to sample type. Letters superscripts in the same file differ significantly (a-b; c-d; e-f; h-i). T0 = during the first week after abortion or delivery, before the primary vaccination; T1 = 2 months after the second dose of primary vaccination; G1 = group 1 (aborted females); G2 = group 2 (normal-delivery females); G3 = group 3 (males). ^1^Prevotellaceae family. *p-value < 0.05; **p-value < 0.01; ***p-value < 0.001.

The bacterial genus and species with the highest RA from each group and sample at T0 and T1 are described in Figures 6, 7. Moreover, significant differences in bacterial genera and species RA observed across female groups and males over time are described in Figure 8. In blood samples (Figure 6A), *Escherichia-Shigella* for G1 (RA = 5%), *Anaplasma* for G2 (RA = 15%), and *Clostidria UCG-014* for male samples (RA = 15%) were the most abundant genera at T0. Anecdotally, *Listeria* was identified in G2 blood samples (RA < 1%) and in males (RA = 1%) at T0. The species taxonomy revealed *Roseomonas gilardii* (RA = 2%), *Curvibacter gracilis* (RA = 2%) for G1, and *Anaplasma marginale* (RA = 15%), together with *Fusobacterium necrophorum* (RA = 3%) for G2, as the most abundant at T0. After primary vaccination (T1) in blood female samples, *Staphylococcus* (G1 RA = 9%; G2 RA = 14%) and *Streptococcus* (G1 RA = 4%; G2 RA = 5%) were the most abundant genera. The dominant species in female samples at T1 were *C. gracilis* (RA = 1%) G1, being greater in G2 samples at T1 (*p* < 0.05; Figure 8E). In G2 samples after primary vaccination (T1), *Streptococcus salivarius* (RA = 3%) represented the most abundant bacterial species, showing increased RA at T1, particularly in G2 samples compared with G1 at T1 (*p* < 0.05; Figure 8E). In bucks, the genus *Mycoplasma* and the species *Mycoplasma ovis* were the dominant taxa at T1 (RA = 82%). In reference to fecal taxonomic description (Figure 6B), *Bacteroides* was the most abundant in G1 at both times (T0 RA = 9%; T1 RA = 8%), being statistically greater compared to G2 at T0 (*p* < 0.05; Figure 8A). This genus was also dominant in the buck microbiota at T0 (RA = 9%). *Treponema* was the dominant genus in G2 feces at T0 (RA = 9%), and *Akkermansia* showed the greatest RA for G2 and males (RA = 8%) at T1. No bacterial species in fecal samples exceed a RA > 1%.

Nasopharyngeal samples showed *Moraxella* as the most abundant genus in all animals and times (T0 media females RA = 59%; T0 media males RA = 29%; T1 media females RA = 48%; T1 media males RA = 31%; Figure 7A). Regarding bacteria species dominance in G1 samples, *Moraxella boevrei* at T0 (RA = 8%), which was statistically higher than in G2 samples (*p* < 0.05; Figure 8B), and *Streptococcus pluranimalium* at T1 (RA = 9%), showed the highest RA. On the contrary, *Mesomycoplasma ovipneumoniae* dominated the respiratory G2 microbiota over time (T0 RA = 8%; T1 RA = 9%), with a greater RA than G1 samples at T1 (*p* < 0.05; Figure 8B). In male nasopharyngeal samples, *M. ovipneumoniae* (RA = 27%) at T0 and *S. pluranimalium*, along with *Mannheimia haemolytica* (RA = 5%), showed the highest RA at T1 microbiota. Anecdotally, it was identified the species *Chlamydia pecorum* in female samples at T0 and T1 (RA < 1%). Related to milk samples (Figure 7B), the genus with the highest abundance for G1 was *Staphylococcus* (T0 RA = 12%; T1 RA = 14%) and *Mannheimia* (RA = 12%) for G2 samples at T0, the latter genus significantly more abundant compared to G1 (*p* < 0.05; Figure 8D). Regarding bacterial species before vaccination (T0), *Corynebacterium crudilactis* (RA = 2%) for G1 and *Staphylococcus simulans*, as well as *Corynebacterium maris* (RA = 2%) for G2, were the most abundant. At T1, *Lactococcus* (RA = 11%), identified as *Lactococcus lactis*, were the dominant taxa for G2 dairy samples, with a significantly higher RA than G1 group (*p* < 0.01; Figure 8D). At this time, the milk bacterial species with the highest RA in G1 samples was *C. maris* (RA = 3%).

Taxonomic vaginal description described at Figure 7C identified *Streptobacillus* (G1 RA = 16%; G2 RA = 30%), *Histophilus* (G1 RA = 11%; G2 RA = 11%), *Ureaplasma* in G1 (RA = 10%) and *Fusobacterium* in G2 (RA = 16%) as the most abundant genera at T0. Prior to vaccination (T0), *Fusobacterium* and *Streptobacillus* were more abundant in G2 samples, and *Streptococcus* in G1 samples (*p* < 0.05; Figure 8C). At T1, the genus *Streptococcus* (G1 RA = 13%; G2 RA = 16%) dominated the vaginal microbiota, followed by *Bacteroides*, and *Ureaplasma* in G1 (RA = 8%), and *Histophilus* (RA = 13%), along with *Escherichia-Shigella* (RA = 10%) in G2 samples. The most prevalent bacterial species identified at T0 were *Clostridium perfringens* in G1 (RA = 10%) and *F. necrophorum* (RA = 14%) in G2. The latter bacterial species showed a significantly decreased RA at T1 in both female groups (*p* < 0.01; Figure 8C). At T1, *S. pluranimalium* (G1 RA = 13%; G2 RA = 16%) showed the highest RA for T1 samples. Finally, *Porphyromonas* (T0 RA = 11%; T1 RA = 14%), and the species *Leptotrichia* sp. (T0 RA = 7%; T1 RA = 6%) were the dominant taxa in buck’s preputial samples. In male reproductive microbiota, the RA of *Oceanivirga, Helcococcus, Corynebacterium,* and *Bacteroides* increased significantly at T1 compared to T0 (*p* < 0.05; Figure 8C).

The genus *Coxiella* and the species *Cb* were identified through metabarcoding in G1 blood at T0 and in milk together with nasopharyngeal samples from both groups, displaying a higher RA in G1 (RA < 1%), and showing a significant decrease at T1 compared to T0 in dairy samples in both female groups (*p* < 0.05; Figure 8D). Metabarcoding revealed the presence of the pathogen in the nasopharynx of males in both time points (RA < 1%), with a lower RA at T1. The pathogens’ presence in vaginal samples revealed a higher abundance of *Cb* in G1 group vaginal samples (RA = 5%) compared to G2 (RA < 1%) at T0 (*p* < 0.05). Moreover, a decreased RA of *Cb* was observed for both groups at T1 (*p* < 0.05; Figure 8C).

#### 3.2.4 General taxonomic composition at T2

**Figure 9:**
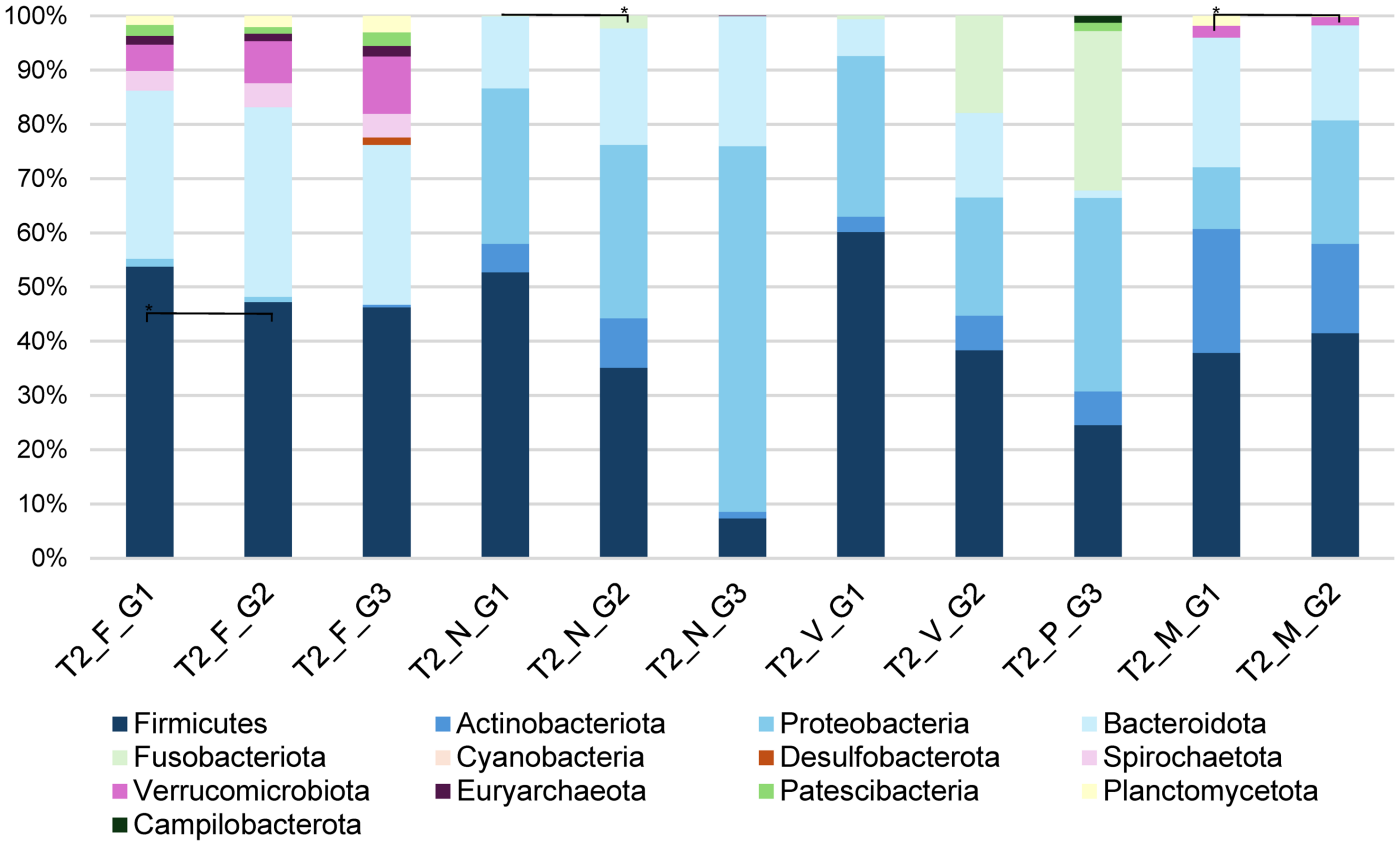
Relative abundance (RA) of taxa at the phylum level of female and male samples at T2, within 1 week postpartum of the next kidding (10 months after the primary vaccination and before revaccination). Only taxa with mean RA > 1% are shown. G1 = group 1 (aborted females); G2 = group 2 (normal-delivery females); G3 = group 3 (males). *p-value < 0.05 (asterisk position indicates the most abundant phylum by group and time).

The RA of the main phyla and significant RA differences among taxa in fecal, nasopharyngeal, and milk samples are shown in Figure 9. Concerning phylum-level taxa, Firmicutes was the most abundant phylum across female fecal, milk, vaginal, and nasopharyngeal samples. In contrast, Proteobacteria dominated nasopharyngeal and preputial samples for males at T2. The phylum Fusobacteriota in G2 nasopharyngeal samples, Verrucomicrobiota for G1 milk samples, and Firmicutes in G1 fecal samples were significantly greater (*p* < 0.05; Figure 9).

**Figure 10:**
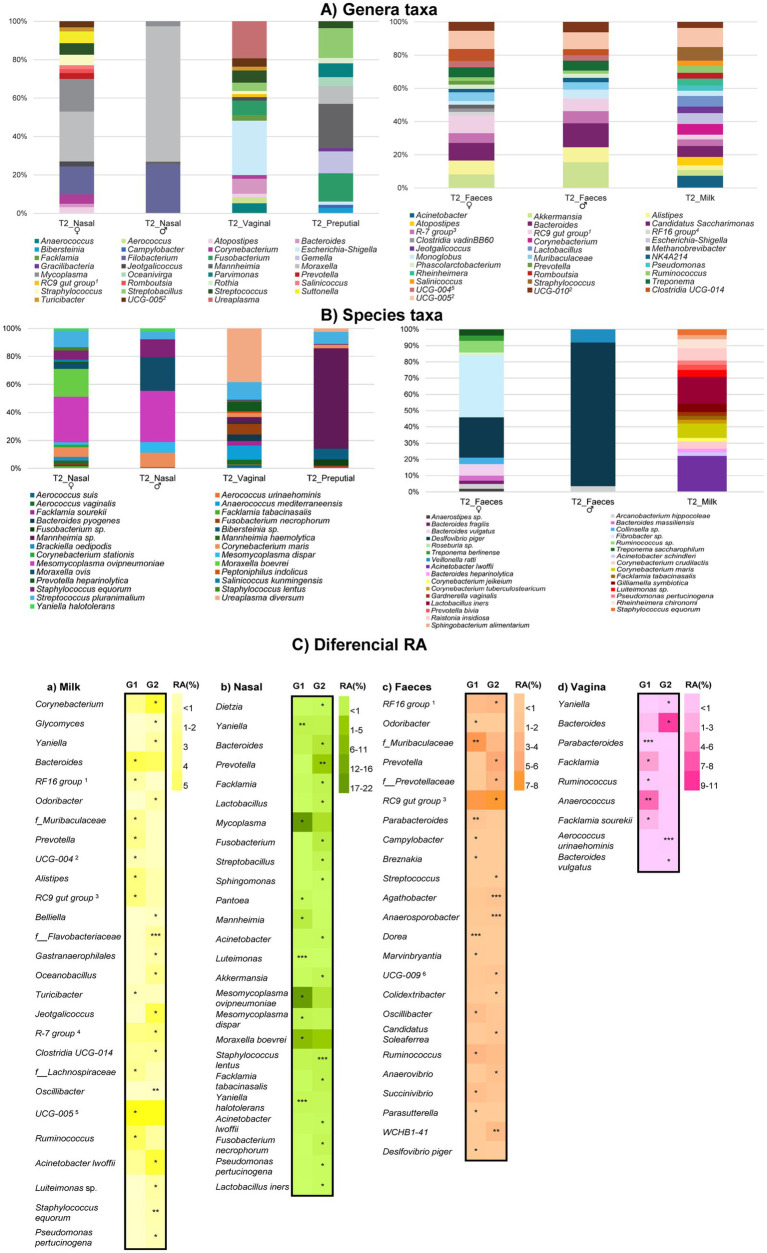
Relative abundance (RA) of taxa at the genus **(A)** and species **(B)** level of female and male samples at T2, within 1 week postpartum of the next kidding (10 months after the primary vaccination and before revaccination). Only taxa with mean RA > 1% are shown. Heat map **(C)** indicates significantly different bacterial genera and species RA across female groups at T2 according to sample type. Asterisk placement indicates samples with higher RA. *f =* family. ^1^Rikenellaceae family; ^2^Oscillospiraceae family; ^3^Christensenellaceae family; ^4^Bacteroidales family; ^5^Prevotellaceae family. ^6^Butyricicoccaceae family. *p-value < 0.05; **p-value < 0.01; ***p-value < 0.001.

Bacterial genus and species with the highest RA at T2 are described in Figure 10. First, the genus and species *Cb* were undetectable in all samples analyzed at T2. Among fecal samples, *UCG-005* in female samples (RA = 7%) and *Akkermansia* (RA = 10%) in males were the dominant genera. No fecal bacterial species exceeded 1% RA. Respiratory samples at T2 indicated that *Moraxella* and *M. ovipneumoniae* were the dominant taxa for females (1 RA = 9% and RA = 12%, respectively) and males (RA = 64% and RA = 2%, respectively). The latter bacterial species was greater in G1 samples (*p* < 0.05). Dairy microbiota showed *UCG-005* (RA = 4%) and *Staphylococcus* (RA = 3%) as the most abundant genera, with *Lactobacillus iners* being the dominant species (RA = 2%). Reproductive microbiota results showed the genera *Escherichia-Shigella* (RA = 22%) and *Ureaplasma* (RA = 15%) as the most prevalent in vaginal samples, as well as *Mannheimia* (RA = 19%) in preputial samples. To conclude, *Ureaplasma diversum* in the vagina (RA = 15%) and *Mannheimia* sp. in the prepuce (RA = 19%) dominated the reproductive microbiota at T2.

#### 3.2.5 General taxonomic composition: bacterial taxa description of environmental samples over time (T0-T2)

The phylum Proteobacteria was the most abundant at T0 in feeding trough samples (RA = 22%), domestic chicken feces (RA = 34%), and in the rubber hose swabs from the milking machine (RA = 50%) at T1. Related to T1 samples, Actinobacteriota in domestic chicken (RA = 20%) and dog’s feces (RA = 49%) presented the highest RA. Finally, Firmicutes in feeding trough (RA = 43%) and bedding samples (RA = 40%) were the most prevalent after vaccination (T1). In T2 samples, Firmicutes was the dominant phylum for bedding (RA = 41%), feeding trough (RA = 54%), dog’s feces (RA = 48%), and rubber hose swabs (RA = 63%). Proteobacteria were the most abundant phylum in chicken feces samples (RA = 28%) at T2 (Supplementary Figure 1).

**Figure 11:**
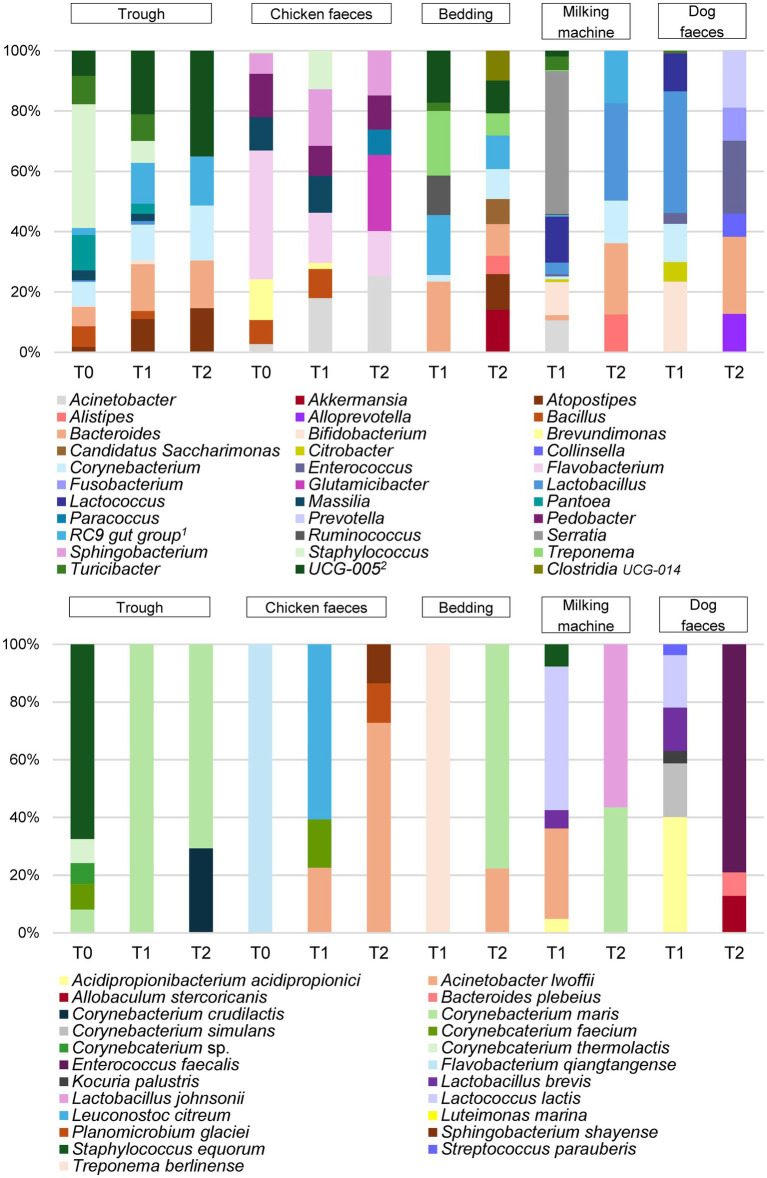
Relative abundance (RA) of taxa at the genus and species level of environmental samples over time. Only taxa with mean RA > 3% for the genera and > 1% for species are shown. T0 = during the first week after abortion or delivery, before the primary vaccination; T1 = 2 months after the second dose of primary vaccination; T2 = within 1 week postpartum of the next kidding (10 months after the primary vaccination and before revaccination). ^1^Rikenellaceae family; ^2^Oscillospiraceae family.

The most abundant genera and species in environmental samples are shown in Figure 11. *Staphylococcus* (RA = 14%) and *S. equorum* (RA = 10%) were the most abundant taxa at T0 feeding trough samples. On the contrary, *UCG-005* (T1 RA = 5%; T2 RA = 9%) and *C. maris* (T1 RA = 2%; T2 RA = 3%) were the dominant at T1 and T2. Chicken fecal samples showed *Flavobacterium* (RA = 16%) at T0, *Sphingobacterium* (RA = 3%) at T1, and *Acinetobacter* (RA = 9%) at T2 as the most abundant genera identified. In these samples, *Flavobacterium qiangtangense* (RA = 1%) at T0, *Leuconostoc citreum* (RA = 7%) at T1, and *A. lwoffii* (RA = 7%) at T2 were the dominant species. Concerning T1 and T2 samples, the most prevalent taxa for the bedding sample were *Bacteroides* (RA = 9%) and *Treponema berlinense* (RA = 1%) at T1, and *Akkermansia* (RA = 6%) and *C. maris* (RA = 4%) at T2. For the rubber hose samples from the milking machine, *Serratia* (RA = 35%) and *L. lactis* (RA = 11%) at T1, and *Lactobacillus* (RA = 8%) and *Lactobacillus johnsonii* (RA = 2%) at T2, were the dominant taxa. Finally, *Lactobacillus* (27%) and *Acidipropionibacterium acidipropionici* (RA = 18%) at T1, and *Bacteroides* (RA = 14%) and *Enterococcus fecalis* (RA = 13%) at T2 were the dominant taxa in dog fecal samples. After primary vaccination (T1), *Bifidobacterium* and *Lactobacillus* increased their RA in the general environmental samples compared to T0 (*p* < 0.01). The genus and the species *Cb* were only identified in the feeding trough sample at T0 (RA < 1%).

## 4 Discussion

This study was motivated by the initial reports of negative impact on the microbiota of aborted goats and sheep belonging to herds with *Cb*-circulating (Toledo-Perona et al., 2025), as well as infection persistence in flocks without early whole-herd vaccination (Zendoia et al., 2024). The present study assessed, for the first time, the progression of *Cb* infection, together with individually caprine and environmental microbiota dynamics in a dairy goat herd after a Q fever outbreak over two consecutive kidding seasons post-implementation of an early and entire-herd coxiellosis vaccination program. Notably, this study represents the first longitudinal characterization of the whole caprine microbiota using metabarcoding.

### 4.1 Normal-delivery goats and bucks contribute to *Cb* persistence and transmission

Despite the fact that different ELISA kits are available to diagnose coxiellosis in ruminants with different specificities and sensitivities (Lurier et al., 2021), the bacteria shedding in seronegative females and males (Table 1) highlights discordance between serological and shedding profiles, consistent with previous studies (Bellini et al., 2014; Joulié et al., 2015; Toledo-Perona et al., 2025). Thus, disease prevalence may be underestimated, increasing transmission risk (Berri et al., 2005; Guatteo et al., 2011), underscoring the need to combine diagnostic tools to accurately assess infection status in small ruminant flocks. In addition, no significant differences in *Cb* shedding were observed among aborted and normal-delivery females, as previously reported (van den Brom et al., 2015a; Toledo-Perona et al., 2025). Additionally, positive preputial and nasopharyngeal samples from a seronegative buck elucidate the important role of males in the epidemiology of the disease. The presence of the bacteria in nasopharyngeal samples of bucks has been reported previously (Toledo-Perona et al., 2025). Nevertheless, this constitutes the first report of *Cb* detection in the buck reproductive tract, although it has previously been documented in rams (Debeljak et al., 2018; Wolf et al., 2020). This finding highlights a previously unrecognized potential risk of *Cb* transmission, suggesting the need for further studies to assess this concern. These epidemiological implications of males and normal-delivery females underscore the importance of including them in control and prevention strategies against Q fever.

### 4.2 Early whole-herd vaccination as a control infection strategy

Several studies have reported the benefits of vaccination in goat herds as a coxiellosis control measure, including high seroconversion and significant reduction in abortion rates and bacterial shedding in milk, feces, and vagina under experimental conditions (Arricau-Bouvery et al., 2005) and natural conditions (Rousset et al., 2009a; Hogerwerf et al., 2011; de Cremoux et al., 2012b; Sting et al., 2013; Boarbi et al., 2014; van den Brom et al., 2015b; Jansen et al., 2022). Concerning serological and qPCR results before vaccination (T0), our results show a seropositivity rate exceeding 70%, consistent with previously reported after clinical outbreaks in caprine herds (Rousset et al., 2009b; Álvarez-Alonso et al., 2018; Miller et al., 2024; Toledo-Perona et al., 2025). The pathogen was detected by qPCR in 100% of the females and in 50% of males. After complete seroconversion of the animals, a significant reduction of more than 60% of animal shedders was observed at T1 (*p* < 0.05). Similar results about short-term vaccination in the serological response and shedding reduction effect in ewes after the booster vaccination (Eibach et al., 2013) and in goats under experimental conditions (Arricau-Bouvery et al., 2005) were observed. During the two subsequent kidding seasons after the primary vaccination (T2 and T3), females’ seropositivity approached 90%, and *Cb* was not detected in any of the sampled animals by qPCR or metabarcoding. It is known that vaccine-induced immunity in goats lasts about 12 months, and without boosters, previously vaccinated animals may resume bacterial shedding (Boarbi et al., 2014). In this context, the seroreversion observed in males at T2 and some females at T3 may pose a risk of reinfections within the herd. Moreover, it is widely established that the optimal strategy involves the vaccination of young uninfected animals (de Cremoux et al., 2012b; Jansen et al., 2022), since infected goats continue shedding the pathogen in milk for up to 2 years following the initiation of vaccination (van den Brom et al., 2013). In this regard, achieving an optimal immunity level status might require adherence to proper vaccination schedules, vaccine handling and administration, and reinforcement through hygiene and control measures. Although abortions may still occur in goats after the initial outbreak (Berri et al., 2007), in our study, abortive episodes have not been reported after primary vaccination. Although it was not an objective of the present study, the genogroup-specific virulence of *Cb* should be taken into account (Long et al., 2019), representing a limitation of this work.

Regarding whole-flock vaccination, in cattle, it has already been suggested that vaccination of at least 80% of the herd is necessary to significantly reduce *Cb* shedding (Taurel et al., 2014). Nevertheless, leaving *Cb*-infected animals unvaccinated under natural conditions would be irresponsible from a public health perspective (Bauer et al., 2021). Studied focus solely on the primiparous female vaccination reported vaginal bacterial shedding, even up to a year post-vaccination (Bauer et al., 2022b; Böttcher et al., 2022) despite high seroconversion rates (Sting et al., 2013). Longitudinal studies have shown that sporadic primiparous female vaccination has reported the persistence of *Cb* for over 10 years (Böttcher et al., 2022). Moreover, a caprine 7-year longitudinal study with replacement-only vaccination showed no reduction in shedding prevalence, with significantly higher levels of fecal and vaginal shedders persisting up to 3 years post-vaccination, and nearly 50% of goats remaining seronegative (Zendoia et al., 2024). Some studies emphasize vaccination as essential to prevent and control Q fever outbreaks in dairy goat farms, highlighting the need for continued application over 5–8 years to achieve pathogen eradication (van den Brom et al., 2015a; Bontje et al., 2016). Our results suggest that early and entire-herd vaccination is a viable medium-long term approach, as it could potentially reduce the risk of environmental contamination and transmission of the disease between individuals.

### 4.3 Nasopharyngeal sample as a potential tool for monitoring coxiellosis in goats

In relation to bacterial presence before vaccination (T0), *Cb* was most detected in the nasopharynx, followed by fecal, vaginal, and milk samples. The nasal route has previously been shown to have high sensitivity and detection rate in goats after a Q fever outbreak (Toledo-Perona et al., 2025). To date, ovine nasal samples have been suggested as indicators of environmental contamination (Bauer et al., 2020) with a limited diagnostic value for monitoring infection dynamics in sheep (Böttcher et al., 2022). The detection of *Cb* in the lungs of goats (Sánchez et al., 2006; Toledo et al., 2023) and experimentally infected kids (Roest et al., 2012) may explain the fact that nasal samples were the most frequently positive and exhibited the greatest persistence throughout the study (Figure 2). At T1, five females remained positive in nasopharyngeal samples and one in milk (Table 1). Although inhalation is widely recognized as the main *Cb* transmission route, continued research on its aerogenic dynamics is still required (Bauer et al., 2023). In addition, a reported evidence described that intranasal experimental infection caused systemic and reproductive consequences in goats (Roest et al., 2012). Despite the need for additional studies in other herds, the present results underscore the nasal sample as the most persistent over time, highlighting the potential of nasopharyngeal sampling for coxiellosis monitoring in goats.

Fecal *Cb* detection was observed in 100% of females at T0. The importance of the fecal route has been reported previously in natural and experimental infections (Arricau-Bouvery et al., 2003; Rodolakis et al., 2007; Roest et al., 2012; Zendoia et al., 2024), reported as one of the most persistent shedding routes in caprine (Bauer et al., 2020). In contrast, other studies have described milk shedding as the main relevant, persistent, and intermittent shedding route in goats (Anastácio et al., 2022; Miller et al., 2024). Our results showed an anecdotal detection of the pathogen in dairy samples, similar to prior reports in *Cb*-infected flocks (Toledo-Perona et al., 2025). Zendoia et al. (2024) reported that prior to vaccination, all sampled goats shed *Cb* through these three routes. However, milk shedding was consistently lower and declined more rapidly compared to other routes. In contrast, after a severe Q fever caprine outbreak, milk excretion was identified as the least frequent route of shedding (Toledo-Perona et al., 2025), consistent with our results. These findings suggest that the udder *Cb*-colonization could be variable in goats. Related to vaginal swabs, over 60% of females were positive, being more frequent in aborted ones. As previously studied, the vaginal shedding after abortion could be higher compared to normal-delivery females (Rousset et al., 2009b). Given the variability of vaginal and dairy shedding, combining sample types may be necessary for accurate coxiellosis diagnosis in goats, as previously suggested by other authors (Miller et al., 2024; Toledo-Perona et al., 2025). Finally, compared to prior studies (Böttcher et al., 2022; Miller et al., 2024; Zendoia et al., 2024), our results show that these shedding periods were shortened when early vaccination was implemented across the entire herd.

### 4.4 Previously unrecognized environmental sources of contamination in caprine dairy herds

This study has identified novel sources of infection in goat herds, specifically rubber hoses from milking machines, feeding troughs, and fly traps from the milking parlor, which had not previously been considered, despite some reports identifying the risk of *Cb* transmission through dust in the milking parlor (Bauer et al., 2022b). Prior studies have demonstrated the pathogen’s environmental presence for at least 2 years in sheep and goat herds (Avberšek et al., 2019; Bauer et al., 2022b; Zendoia et al., 2024), implying a sustained risk of transmission to animals and humans. Before vaccination, bedding samples from the kidding area and feeding troughs samples were positive by qPCR, consistent with findings in caprine infected herds (Toledo-Perona et al., 2025). These authors attributed feeder contamination to the presence of *Cb* in the nasopharynx, in line with our results. Accordingly, the present study reports *Cb* presence in milking parlor metal feeders, and we propose flame-torching as a potential method for *Cb* spores elimination. In relation to milking parlor contamination, qPCR positive samples from dead flies from an insect trap, may reflect the persistence of viable *Cb* in dust (Álvarez-Alonso et al., 2020) and their potential role as a vehicle for pathogen spread among animals. In this regard, further studies would be required to determine whether these findings reflect a generalized pattern or are instead associated with sporadic Q fever outbreak events.

At T1, although *Cb* was anecdotally detected in milk, the pathogen was identified in rubber hose swabs from the milking machine degraded. This fact suggests a previously unrecognized source of *Cb*-contamination and reinforces the hygienic importance of the milking machine maintenance. Continuing with T1, bedding and troughs samples remained positive, revealing deficiencies in hygienic and disinfection control measures. No positive environmental samples were detected in the subsequent two kidding seasons (T2 and T3). The fact that the pathogen was not detected by qPCR and metabarcoding techniques at this time suggests that the environmental persistence of *Cb* can be controlled in the short term when early and whole-herd vaccination, together with enhanced farm hygienic, cleaning, and disinfection practices, are implemented. These findings are consistent with (Álvarez-Alonso et al., 2018), who reported that although *Cb* remained viable following abortions, no viable bacteria were detected in the dust 2 months after the implementation of the control measures. Regarding domestic animal implications, chicken feces samples were positive at T0, as previously reported in that animal species (Muramatsu et al., 2006; Tatsumi et al., 2006). Nevertheless, this fact had not previously been reported in sheep herds with less severe Q fever outbreaks (Toledo-Perona et al., 2025). This finding may indicate that the severity of the outbreak may increase the exposure of other animals, increasing the surrounding environmental contamination. Moreover, the study of the fecal microbiota of domestic chicken confirmed the absence of *Cb* after vaccination, consistent with the findings observed in the qPCR results. However, the exclusion of domestic chickens within a herd should always be a biosecurity measure implemented to avoid environmental contamination.

### 4.5 Restoration of taxonomic evenness regardless of abortion

The present study represents the first individual monitoring of the whole caprine microbiota across two kidding seasons after a Q fever outbreak, followed by a whole-herd vaccination program implementation. Microbiota composition differed between female groups over time in all anatomical locations studied (Figures 3, 4). Alpha diversity results indicated that in both female groups, fecal microbial richness increased after vaccination, and in nasopharyngeal samples for G2 samples at T2 (*p* < 0.05). This fact could be associated with the absence of clinical signs, infection pressure reduction. Moreover, the ruminant digestive microbiota can be influenced by climate and environmental conditions, including the health status of the host (Clavel et al., 2017; Liu et al., 2021). In this sense, in pigs and cattle, higher microbiota richness has been linked to increased respiratory stability and resistance to pathogens (Li et al., 2021; Centeno-Martinez et al., 2022), improving digestive efficiency and health in bovines (Welch et al., 2020). Moreover, Miao et al. (2023) reported significantly reduced lung microbial diversity in sheep exhibiting pneumonia compared with asymptomatic animals. However, a lower richness respiratory microbiota at T1 compared to T0 was observed (*p* < 0.01), with respiratory G1 richness greater at T0 (*p* < 0.05). Similar to observations in cows, abortion can alter the normal uterus environment and favor the expansion of opportunistic communities, oxidative stress, and neutropenia (Amin et al., 2023). Taking into account the potential respiratory tropism of *Cb* (Toledo-Perona et al., 2025) and pneumonia associated (Martinov et al., 1989; Wouda and Dercksen, 2007) in small ruminants, microbial dysbiosis mentioned in the reproductive tract may occur similarly in the upper respiratory tract. Furthermore, direct contact during the postpartum maternal licking behavior of aborted fetuses may facilitate nasal colonization by diverse bacterial communities. Following the evenness index results, despite being lower at T1 compared to T0 (*p* < 0.05), the dairy microbiota of G2 showed a higher microbial uniformity (*p* < 0.05). After vaccination (T1), this index increased in prepuce microbiota compared to T0 (*p* < 0.01). Nevertheless, increased uniformity does not always reflect a healthy microbiota, as it may indicate loss of dominant keystone species, as described in the milk of small ruminants after abortion occurrence (Toquet et al., 2021). This suggests a dairy and respiratory dysbiosis following Q fever outbreak, possibly linked to a compromised immune status, that persisted even after primary vaccination.

Beta diversity results highlighted significant structural differences between female groups across all anatomical locations, except for milk, between T0 and T1 (Figure 4). The results may indicate that the reproductive consequences induce short-term microbial restructuring in fecal, nasopharyngeal, vaginal, and blood microbiota. Nasopharyngeal and vagina microbiota at T0 differed significantly between groups (*p* < 0.05), supporting a potential localized dysbiosis after the outbreak. Particularly, beta diversity different patterns observed in the vagina are in coherence with previously reported small ruminant reproductive microbiota impact after Q fever outbreaks (Toledo-Perona et al., 2025). In the same study, no differences in alpha diversity were observed between aborted and normally delivered goats, which is consistent with our findings. Thus, severe clinical abortion outbreaks can impact reproductive microbial richness and evenness regardless of abortion occurrence. Finally, microbial diversity differences in fecal and nasopharyngeal samples related to abortion status may be less evident at T2, showing an overall microbiota recovery. Further research is required to determine the productive implications of this microbial instability and to assess outcomes without control measures and the vaccination impact.

### 4.6 Firmicutes as a whole health biomarker: dominance after vaccination

Several findings indicate a key role of Firmicutes in the restoration of the whole microbiota following a Q fever outbreak. A shift in Firmicutes dominance was observed at T1, with increased RA in the vaginal, preputial, fecal, and blood microbiota, becoming significantly higher in fecal and vaginal samples of G2 at this time (*p* < 0.05). In contrast, Proteobacteria dominated the blood microbiota at T0, consistent with previously reported findings in aborted ewes, where Firmicutes prevailed in normal-delivery females (Toledo-Perona et al., 2025). This phylum shift may suggest microbial recovery at T1, as genera with Proteobacteria (e.g., *Escherichia-Shigella*, *Sphingomonas,* and *Coxiella*) decreased, while Firmicutes-associated taxa (e.g., *Staphylococcus, Streptococcus*, *Lactobacillus, and Anaerococcus*) increased. In feces, Bacteroidota was dominant at T0, aligning with prior findings after Q fever outbreak occurrence (Toledo-Perona et al., 2025). Concerning T1 and T2, Firmicutes was the dominant phylum, consistent with previous reports in small ruminant fecal microbiota (Tanca et al., 2017; Zhang et al., 2018). Pertaining to milk samples, Firmicutes remained dominant throughout the study, consistent with reports in sheep and goats (Toquet et al., 2021), and with normal-delivery females in *Cb*-infected herds (Toledo-Perona et al., 2025). Finally, Firmicutes was the most abundant phylum across all anatomical locations at T2.

Fusobacteria in the vagina and Proteobacteria in the preputial samples predominated at T0. Both phyla included pathogens linked to reproductive disorders in domestic ruminants (Bicalho et al., 2017; Poole et al., 2023; Reinoso-Peláez et al., 2025). On the contrary, Firmicutes dominated vaginal microbiota after vaccination (T1 and T2). This phylum has been reported in preputial samples and is associated with pregnant and nulliparous ewes (Serrano et al., 2020; Koester et al., 2021; Reinoso-Peláez et al., 2023; Barba et al., 2024; Cassas et al., 2024; Toledo-Perona et al., 2025). Finally, Proteobacteria dominated the nasopharyngeal microbiota at T0 and T1, with Firmicutes being the dominant phylum at T2. A previous association between Proteobacteria dominance and high nasopharyngeal detection of *Cb*, species included in this bacterial phylum, has been performed (Toledo-Perona et al., 2025). Moreover, Miao et al. (2023) showed that pneumonic sheep lungs exhibit a higher abundance of Proteobacteria and Fusobacteria, whereas Firmicutes predominated in healthy lungs’ microbiota These shifts may reflect changes in the respiratory microbiota during the outbreak and its subsequent stabilization, supporting the previously described role of Firmicutes in microbial homeostasis (Li et al., 2021).

### 4.7 *Cb* vaginal load and bacteriemia as drivers of abortion in goats

This study reports the novel detection of *Cb* in blood and nasopharyngeal samples of goats using metabarcoding analysis. At T0, *Cb* was identified in vaginal, milk, and nasopharyngeal samples from both groups, and in G1 blood samples. Notably, the pathogen was also detected in buck respiratory samples at T0 and T1, which may indicate subclinical infection, consistent with the seroreversion observed at T2 and T3. Detection of *Cb* exclusively in G1 blood samples suggests a possible bacteriemia occurrence, which could contribute to the occurrence of abortion. Metabarcoding revealed a higher RA of *Cb* in vaginal samples of G1 (5%) compared to G2 (0.2%) at T0 (*p* < 0.05). Previously, reports indicated a higher *Cb* load shed after abortion compared with normal-delivery females by PCR (Joulié et al., 2015). A RA < 1% of *Cb* was reported in the vaginal microbiota of infected non-vaccinated sheep and goats (Toledo-Perona et al., 2025). These findings may elucidate that reproductive bacterial load and/or bacteriemia may act as a trigger for abortion in *Cb-infected* goats. Following vaccination, a complete *Cb* RA reduction was observed in milk and vaginal samples (*p* < 0.05; Figure 8), highlighting a possible microbial composition transition after control measures implementation maintained through the subsequent kidding season.

### 4.8 Taxonomic fluctuations after vaccination and a shift toward beneficial profiles

Regarding female blood microbiota at T0, a higher RA of *A. marginale*, *Fusobacterium* o *M. ovis*, taxa associated with immunosuppressive processes in small ruminants (Machado et al., 2017; Ceylan et al., 2021), may have influenced the clinical infection course. In contrast, lactic acid bacteria (LAB) genera increase (*Streptococcus*, *Lactobacillus,* and *Alloiococcus*) at T1, which could suggest a recovery of the systemic microbiota following vaccination. Specifically, *Anaplasma* and *Pseudomonas* dominated the microbiota for G2 samples at T0, consistent with findings in sheep and rams under a semi-extensive system (Toledo-Perona et al., 2025). At T1, *Staphylococcus, Streptococcus,* and *Pelomonas* were the most prevalent genera, also reported in blood caprine samples after a Q fever outbreak (Toledo-Perona et al., 2025). Within LAB presence at T1, *S. sali*var*ius* decreased in G1 and increased in G2 (*p* < 0.05), bacterial species with potential probiotic relevance in humans (Al-Akel et al., 2024). Finally, *L. johnsonii* shifted from higher RA in G2 at T0 (*p* < 0.05) to higher RA in G1 at T1 (*p* < 0.05). This species is linked to gut health in sheep and calves (Fernández-Ciganda et al., 2022; Wang et al., 2025). Nevertheless, the presence of these bacterial communities observed in the blood microbiota in our study may instead reflect the transient and sporadic bacterial translocation between different anatomical body sites that has been reported in humans (Tan et al., 2023).

In relation to fecal samples, the genus *Bacteroides* and *RC9 gut group* (Rikenellaceae family) were consistently prevalent, in line with previous reports (Domínguez et al., 2022; Toledo-Perona et al., 2025). The significant RA of *UCG-003* (Prevotellaceae family) in G2, associated with healthy fecal ovine microbiota (Zeng et al., 2017; Mamun et al., 2020), or the increase of *Treponema* at T1 in aborted goats, described in normal-delivery females under *Cb* circulation (Toledo-Perona et al., 2025), may suggest microbial restoration. With respect to nasopharyngeal microbiota, while *Lactobacillus* has been reported among dominant pulmonary taxa in sheep (Glendinning et al., 2016) and in nasal samples in ovine herds with less severe *Cb-*infection (Toledo-Perona et al., 2025), it was absent here following a severe outbreak, indicating possible suppression of LAB. Instead, *Staphylococcus, Filobacterium,* and *Mannheimia* were abundant at T0, all previously associated with respiratory samples prevalent in *Cb*-infected small ruminants (Toledo-Perona et al., 2025). *Staphylococcus* and *Mannheimia* were also described in the pulmonary sheep microbiota (Glendinning et al., 2016, 2017). The detection of *M. ovipneumoniae* in the absence of LAB supports the hypothesis that LAB may limit pathogenic mycoplasmas colonization (Toquet et al., 2023). In male samples, *Mycoplasma* dominated at T0, as previously reported in rams from *Cb-positive* herds (Toledo-Perona et al., 2025), which may be associated with more severe pneumonias involving other respiratory pathogens, as previously reported (Dassanayake et al., 2010), including *Cb*, which could account for its detection at T1. Despite this, at T1, the significant reduction of *Mycoplasma* and *M. ovipneumoniae* (*p* < 0.05) could indicate a nasopharyngeal microbial restoration and a potential respiratory microbiota stability. Moreover, the anecdotical detection of *Chlamydia* spp. (RA < 1%) reflects co-infection dynamics under immune stress conditions (Glendinning et al., 2016). In females, results after primary vaccination (T1), a significant reduction of *Moraxella* spp. and *Corynebacterium* spp. was observed in G1 samples, both described in the respiratory microbiota of sheep (Glendinning et al., 2016, 2017). Finally, some LAB, such as *Enterococcus,* increased at T1 in G2 (*p* < 0.05). This genus has been reported in the healthy respiratory ovine microbiota (Glendinning et al., 2016). Nonetheless, it should also be taken into account that the upper respiratory tract microbiota of ruminants can be modulated by factors such as antimicrobial exposure, stress, and the host’s immune status (Chai et al., 2022).

In milk taxonomic results, *Staphylococcus* remained abundant across time points with higher RA in G1 samples. Despite this genus being reported in healthy sheep (Castro et al., 2019; Esteban-Blanco et al., 2020a, 2020b) and caprine milk (Polveiro et al., 2020), its RA was higher in aborted *Cb-*infected sheep (Toledo-Perona et al., 2025), and even associated with mastitis in these animal species (Toquet et al., 2021). This result suggests the occurrence of dysbiosis-associated shifts in the mammary gland following a Q fever outbreak in goats. Pathogens linked to mastitis in sheep and goats (*Mannheimia*, *Turicibacter, Jeotgalicoccus,* and *S. equorum*; Castro et al., 2019; Esteban-Blanco et al., 2020a; Polveiro et al., 2020), were either more abundant in G1 or decreased at T1. In contrast, *Enterococcus* and *Lactococcus* (identified as *L. lactis*) are typically dominant in healthy goat milk (Callon et al., 2007), prevailed in G2 and increased at T1 (*p* < 0.05). Other dominant genera in the dairy microbiota after vaccination (T1 and T2) were *Corynebacterium*, reported as more abundant in normal-delivery ewes (Toledo-Perona et al., 2025), and included in the dairy ovine microbial core (Esteban-Blanco et al., 2020a).

Reproductive microbiota showed the greatest taxonomic variation, underscoring sexual microbiota modulation between both sexes (Barba et al., 2024) and the impact of abortion on the vaginal microbiota composition of *Cb*-infected goats. The taxonomic description, vaginal microbiota at T0 was dominated by *Streptobacillus*, declining at T1 (*p* < 0.01). In sheep, despite this genus being associated with vaginitis occurrence (Toquet et al., 2025), it has been described its dominance in vaginal ovine microbiota (Swartz et al., 2014; Serrano et al., 2020; Reinoso-Peláez et al., 2025). For G1, genera associated with reproductive disorders, such as *Coxiella* (*p* < 0.05), *Ureaplasma,* and *Mannheimia* (Agerholm, 2013; Oliveira et al., 2017; Jesse et al., 2020) were more abundant in G1 samples at T0. Moreover, *Coxiella*, *Streptobacillus*, *Fusobacterium* (*p* < 0.05), *Mannheimia*, and particularly *Histophilus* in G1, together with *Bacteroides* in G2, decreased post-vaccination (T1). Notably, *Ureaplasma* decreased in G1 at T1, remaining prevalent at T2 in both goat groups. This genus dominated the vaginal microbiota of aborted ewes in previous studies (Toledo-Perona et al., 2025), but was also present in ewes with reproductive success (Serrano et al., 2020; Barba et al., 2024). The variation in *Ureaplasma* spp. RA may reflect a yet unexplored role of this genus, with reproductive implications that remain unclear. In G2 vaginal microbiota, *Aerococcus, Anaerococcus,* and *Porphyromonas* were more abundant at T0 compared to G1, as previously described in sheep (Greenwood et al., 2022; Reinoso-Peláez et al., 2025). *Escherichia-Shigella* was also dominant in G2 vaginal samples at T0. The prevalence of this genus has been reported in the vagina of non-pregnant ewes (Barba et al., 2024) and in *Cb-infected* females (Toledo-Perona et al., 2025). Its RA increased at T1, and it became the dominant taxa at T2 in both female groups. Other genera associated with reproductive success in sheep included *Streptococcus, Atopostipes,* and *Aerococcus* (Koester et al., 2021; Barba et al., 2024; Toquet et al., 2025), which also increased at T1 in the overall vaginal microbiota. Finally, preputial microbiota was dominated by *Porphyromonas* at T0 and T1, similar to findings in rams from *Cb*-infected herds (Toledo-Perona et al., 2025). Finally, *Mannheimia* was the dominant taxon at T2, previously described in the ram prepuce (Konak and Avdatek, 2025).

Regarding the metabarcoding results obtained from environmental samples, although these were not assessed longitudinally, the microbiota of domestic animals has previously been described in Q fever outbreaks in small ruminant herds (Toledo-Perona et al., 2025). In the present study, the bacterial communities detected in the feces of domestic animals fluctuated over time, which may indicate a potential influence of the microbial dynamics observed within the herd. Finally, the findings in the caprine microbiota observed in this study suggest a favorable evolution toward increased LAB abundance. This rise was also detected in environmental samples following primary vaccination, indicating that monitoring environmental microbiota may be useful for understanding microbial dynamics in caprine herds.

In conclusion, this study represents the first longitudinal study of the whole caprine microbiota. The present work provides evidence of microbial disruption associated with abortion, characterized by alterations in microbial communities from the vagina, nasopharynx, feces, and blood. Restoration of microbial stability in the subsequent kidding season suggests that immune status and the infection pressure reduction may contribute to the microbiota recovery. The increase of pathogenic taxa in females after outbreak occurrence, higher in aborted ones, highlights possible reproductive and productive implications. Firmicutes emerged as a potential biomarker of microbiota restoration after a Q fever outbreak. Moreover, lactic acid bacteria appeared to contribute to microbial and host recovery. In addition, the association between abortion and both the presence of *C. burnetii* in blood and its significantly elevated abundance in vaginal samples underscores the importance of preventing systemic infections. Regarding coxiellosis control, *C. burnetii* persistence in caprine outbreaks could be reduced when a complete and early vaccination is implemented, which therefore reduces the risk of infection to the human population. Nasopharyngeal PCR testing seems a promising and useful approach for coxiellosis monitoring in caprine flocks. Moreover, the bucks should be included in the control measures against the disease. In this sense, hygienic and biosecurity measures, including disinfection of the milking parlor, are relevant to prevent reinfection and ensure effective disease control in goat herds. Additional studies across diverse herds are required to validate these findings, including further reports concerning microbiota pathogen interactions after Q fever outbreaks in caprine.

## Data Availability

The datasets generated for this study can be found in the European Nucleotide Archive (ENA) (European Molecular Biology Laboratory, European Bioinformatics Institute (EMBL-EBI)): https://www.ebi.ac.uk/ena/browser/home, with the study ID PRJEB95894.
