# Supplementary material for: Impact of whole-herd vaccination on a caprine coxiellosis outbreak: a longitudinal study of Coxiella burnetii shedding, serology, and host microbiota
Source: Front Microbiol. 2026 Jun 3;17:1824800. doi: 10.3389/fmicb.2026.1824800 (PMC13273978; doi:10.3389/fmicb.2026.1824800)
Supplement: Supplementary file 1 [file Table_1.DOCX]

Supplementary Material

**Supplementary Table S1.** Number of phylotypes detected depending on the type of sample and sampling time.

|  |  | T0-T1 | T2 |
| --- | --- | --- | --- |
| Female’s samples | Milk | 2,213 | 802 |
|  | Faecal | 6,368 | 2,414 |
|  | Blood | 473 | -^1^ |
|  | Nasal | 2,161 | 618 |
|  | Vaginal | 3,232 | 462 |
| Male’s samples |  |  | 1,000 |
|  | Faecal | 8,807 |  |
|  | Blood^1^ | 81 |  |
|  | Nasal | 166 |  |
|  | Preputial | 369 |  |
| Environmental samples |  | 2,725 | 712 |

^1^Not sampled at T2. T0 = during the first week after abortion or delivery, before the primary vaccination; T1 = two months after the second doses of primary vaccination; T2 = within one week postpartum of the next kidding (10 months after the primary vaccination and before revaccination).

**Supplementary Table S2.** Median (interquartile interval) of the alpha diversity indices of faecal, milk, blood, vaginal and preputial microbiota studied.

| **Comparation** | **Alpha diversity metric** | | **Faecal** | **Milk** | **Nasal** | **Blood** | **Vaginal/**  **Preputial** |
| --- | --- | --- | --- | --- | --- | --- | --- |
| T0_T1 (G1) | Richness (Observed ASV´s) | *P*-value | 0.00222** | 0.32668 | 0.00006*** | 0.73538 | 0.09908 |
|  |  | T0 | 221.00 | 78.00 | 141 | 21.00 | 65.50 |
|  |  | T1 | 303.00 | 80.00 | 38.50 | 20.00 | 142.50 |
|  | Evenness (Pielou index) | *P*-value | 0.7148 | 0.81276 | 0.00073*** | 0.17144 | 0.33677 |
|  |  | T0 | 0.91 | 0.92 | 0.80 | 0.90 | 0.66 |
|  |  | T1 | 0.92 | 0.88 | 0.59 | 0.881 | 0.83 |
| T0_T1 (G2) | Richness (Observed ASV´s) | *P*-value | 0.00517** | 0.12454 | 0.00837** | 0.4716 | 0.118 |
|  |  | T0 | 203.00 | 78.00 | 84.00 | 10.00 | 21.00 |
|  |  | T1 | 280.00 | 68.00 | 37.50 | 16.00 | 52.00 |
|  | Evenness (Pielou index) | *P*-value | 0.56886 | 0.01862* | 0.03417* | 0.7175 | 0.51003 |
|  |  | T0 | 0.91 | 0.71 | 0.73 | 0.86 | 0.77 |
|  |  | T1 | 0.91 | 0.92 | 0.60 | 0.91 | 0.66 |
| T0_T1 (G3) | Richness (Observed ASV´s) | *P*-value | 0.53336 |  | 0.07482 | -^1^ | 0.53748 |
|  |  | T0 | 335.50 |  | 36.50 | -^1^ | 98.50 |
|  |  | T1 | 283.00 |  | 51.50 | -^1^ | 106.50 |
|  | Evenness (Pielou index) | *P*-value | 0.13229 |  | 0.75537 | -^1^ | 0.00756** |
|  |  | T0 | 0.93 |  | 0.61 | -^1^ | 0.85 |
|  |  | T1 | 0.93 |  | 0.62 | -^1^ | 0.88 |
| G1_G2 (T0) | Richness (Observed ASV´s) | *P*-value | 0.98811 | 0.90303 | 0.02766* | 0.85714 | 0.1761 |
|  |  | G1 | 221.00 | 78.00 | 141 | 21.00 | 65.50 |
|  |  | G2 | 203.00 | 78.00 | 84.00 | 10.00 | 21.00 |
|  | Evenness (Pielou index) | *P*-value | 0.74535 | 0.02084* | 0.11697 | 0.85697 | 0.89209 |
|  |  | G1 | 0.91 | 0.92 | 0.80 | 0.90 | 0.66 |
|  |  | G2 | 0.91 | 0.71 | 0.73 | 0.86 | 0.77 |
| G1_G2 (T1) | Richness (Observed ASV´s) | *P*-value | 0.72641 | 0.9497 | 0.89779 | 0.57256 | 0.15308 |
|  |  | G1 | 303.00 | 80.00 | 38.50 | 20.00 | 142.50 |
|  |  | G2 | 280.00 | 68.00 | 37.50 | 16.00 | 52.00 |
|  | Evenness (Pielou index) | *P*-value | 0.66831 | 0.79644 | 0.98112 | 0.52422 | 0.88333 |
|  |  | G1 | 0.92 | 0.88 | 0.59 | 0.881 | 0.83 |
|  |  | G2 | 0.91 | 0.92 | 0.60 | 0.91 | 0.66 |
| G1_G2 (T2) | Richness (Observed ASV´s) | *P*-value | 0.02029* | 0.60067 | 0.02575* | -^2^ | -^1^ |
|  |  | G1 | 475 | 33 | 102 | -^2^ | 118 |
|  |  | G2 | 412 | 86 | 140 | -^2^ | 83 |
|  | Evenness (Pielou index) | *P*-value | 0.77076 | 0.71664 | 0.16137 | -^1^ | -^1^ |
|  |  | G1 | 0.94 | 0.91 | 0.63 | -^1^ | 0.65 |
|  |  | G2 | 0.93 | 0.92 | 0.79 | -^1^ | 0.67 |

*< 0.05; **< 0.01; ***< 0.001. ^1^Not statistical analysis performed; ^2^Not sampled at T2. ASVs = amplicon sequence variant; G1 = aborted females; G2 = normal-delivery females; G3 = males; T0 = during the first week after abortion or delivery, before the primary vaccination; T1 = two months after the second doses of primary vaccination; T2 = within one week postpartum of the next kidding (10 months after the primary vaccination and before revaccination).

**Supplementary Table S3.** Median (interquartile interval) of the alpha diversity indices of environmental samples.

| Comparison | Alpha diversity metric | | |
| --- | --- | --- | --- |
| T0_T1 | Richness (Observed ASV´s) | *P*-value | 0.40477 |
|  |  | T0 | 551 |
|  |  | T1 | 351 |
|  | Evenness (Pielou index) | *P*-value | 0.54805 |
|  |  | T0 | 0.90 |
|  |  | T1 | 0.91 |

ASVs = amplicon sequence variant; T0 = during the first week after abortion or delivery, before the primary vaccination; T1 = two months after the second doses of primary vaccination.

**Supplementary Table S4.** FDR-adjusted *P* values of pairwise comparisons using PERMANOVA analysis for beta diversity indexes matrices in the three comparisons at T0-T1 comparison (between experimental times T0 *vs*. T1; between experimental groups G1 *vs.* G2; between group and time).

| Type of sample | Group comparison | Qualitative indexes | | Quantitative indexes | | |
| --- | --- | --- | --- | --- | --- | --- |
|  |  | Jaccard | Unweighted UniFrac | | Bray Curtis | Weighted UniFrac |
| Faecal | Females (T0_T1) | 0.118 | 0.002** | | 0.360 | 0.124 |
|  | Females (G1_G2) | 0.566 | 0.005** | | 0.514 | 0.339 |
|  | Females (Group_Time) | 0.921 | 0.664 | | 0.668 | 0.786 |
|  | Males (T0_T1) | 1 | 1 | | 1 | 1 |
| Milk | Females (T0_T1) | 0.358 | 0.215 | | 0.420 | 0.062 |
|  | Females (G1_G2) | 0.049* | 0.712 | | 0.146 | 0.238 |
|  | Females (Group_Time) | 0.408 | 0.936 | | 0.154 | 0.176 |
| Nasal | Females (T0_T1) | 0.001** | 0.001** | | 0.001** | 0.001** |
|  | Females (G1_G2) | 0.001** | 0.001** | | 0.002** | 0.001** |
|  | Females (Group_Time) | 0.288 | 0.325 | | 0.271 | 0.118 |
|  | Males (T0_T1) | 0.3333333 | 0.3333333 | | 0.3333333 | 0.3333333 |
| Blood | Females (T0_T1) | 0.266 | 0.001** | | 0.095 | 0.001** |
|  | Females (G1_G2) | 0.106 | 0.404 | | 0.113 | 0.859 |
|  | Females (Group_Time) | 0.567 | 0.141 | | 0.288 | 0.067 |
| Vaginal | Females (T0_T1) | 0.001** | 0.003** | | 0.001** | 0.001** |
|  | Females (G1_G2) | 0.001** | 0.008** | | 0.001** | 0.001** |
|  | Females (Group_Time) | 0.090 | 0.178 | | 0.309 | 0.090 |
| Preputial | Males (T0_T1) | 0.6666667 | 0.3333333 | | 0.6666667 | 0.6666667 |
| Environment^1^ | (T0_T1) | 0.948 | 0.687 | | 0.633 | 0.786 |

**P* < 0.05; ***P* < 0.01; ****P* < 0.001. G1 = aborted females; G2 = normal-delivery females; T0 = during the first week after abortion or delivery, before the primary vaccination; T1 = two months after the second doses of primary vaccination.

**Supplementary Figure 1.** Relative abundance (RA) of taxa at the phylum level from environmental samples over time. Only taxa with mean RA > 1 % are shown; T0 = during the first week after abortion or delivery, before the primary vaccination; T1 = two months after the second doses of primary vaccination; T2 = within one week postpartum of the next kidding (10 months after the primary vaccination and before revaccination).
